# Parieto-Occipital Electrocortical Dynamics during Real-World Table Tennis

**DOI:** 10.1523/ENEURO.0463-22.2023

**Published:** 2023-04-13

**Authors:** Amanda Studnicki, Daniel P. Ferris

**Affiliations:** J. Crayton Pruitt Family Department of Biomedical Engineering, University of Florida, Gainesville, FL 32611

**Keywords:** electroencephalography, independent component analysis, mobile brain-body imaging, table tennis

## Abstract

Traditional human electroencephalography (EEG) experiments that study visuomotor processing use controlled laboratory conditions with limited ecological validity. In the real world, the brain integrates complex, dynamic, multimodal visuomotor cues to guide the execution of movement. The parietal and occipital cortices are especially important in the online control of goal-directed actions. Table tennis is a whole-body, responsive activity requiring rapid visuomotor integration that presents a myriad of unanswered neurocognitive questions about brain function during real-world movement. The aim of this study was to quantify the electrocortical dynamics of the parieto-occipital cortices while playing a sport with high-density electroencephalography. We included analysis of power spectral densities (PSDs), event-related spectral perturbations, intertrial phase coherences (ITPCs), event-related potentials (ERPs), and event-related phase coherences of parieto-occipital source-localized clusters while participants played table tennis with a ball machine and a human. We found significant spectral power fluctuations in the parieto-occipital cortices tied to hit events. Ball machine trials exhibited more fluctuations in θ power around hit events, an increase in intertrial phase coherence and deflection in the event-related potential, and higher event-related phase coherence between parieto-occipital clusters as compared with trials with a human. Our results suggest that sport training with a machine elicits fundamentally different brain dynamics than training with a human.

## Visual Abstract

**Figure:**
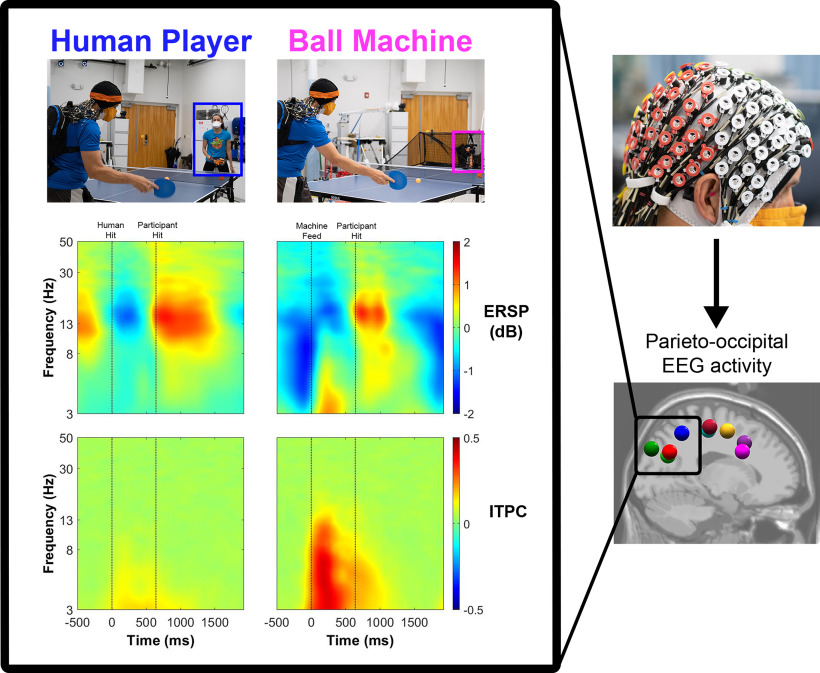

## Significance Statement

Analyzing high-density scalp electroencephalography (EEG) from human participants playing table tennis allowed us to examine the precise timing of electrocortical changes in the parieto-occipital cortices during a whole body visuomotor task with high ecological validity. Time-frequency and connectivity analyses revealed earlier broadband desynchronization, more evidence of phase-locked activity, and an increase in coherence between brain regions in ball machine trials than in human trials. These differences likely reflect how humans interpret body and machine cues regarding the trajectory and speed of the oncoming ball, which may have important implications in sport training.

## Introduction

The parieto-occipital cortices play key roles in the online control of goal-directed movements (Y.E. Cohen and Andersen, 2002; Rizzolatti and Matelli, 2003; Milner and Goodale, 2006; Sereno and Huang, 2014). Research has shown that parieto-occipital functions are necessary for tasks like reaching (Perenin and Vighetto, 1988), grasping (Jeannerod et al., 1995), catching (Machado et al., 2008), and avoiding (McVea and Pearson, 2009) objects. However, most of these studies used controlled environments rather than real-world tasks with high ecological validity (Gramann et al., 2014; Ladouce et al., 2017). Cues from the environment guide sensorimotor behavior since perception and action are tightly coupled processes (Melnik et al., 2017; Yang et al., 2018; McNamee and Wolpert, 2019). Especially when we study a region like the parieto-occipital cortex, the authenticity of the experiment determines our understanding of cognition during movement (Whitlock, 2017). Electroencephalography (EEG) is a particularly good real-world brain imaging technique (Gramann et al., 2011). Typically, a disadvantage of EEG is its susceptibility to artifacts (Urigüen and Garcia-Zapirain, 2015), but recent technological advancements enable mobile EEG recordings (Nordin et al., 2019; Studnicki et al., 2022).

Table tennis is a responsive activity that could provide insight into sensorimotor integration, anticipation, and object interception. The distance between table tennis athletes is small (<3 m), which gives players little time to perceive, plan, hit, and recover from a ball that could be hit at 17 m/s (38 mph; Bootsma and van Wieringen, 1990). Studies have found that elite players have faster reactions, greater hand movement speed, and more efficient visuomotor integration skills than nonathletes (Hülsdünker et al., 2019; Hughes et al., 2021). Additionally, table tennis interventional studies have reduced cognitive decline, promoted executive function, and improved mental acuity (Gu et al., 2019; Zhou et al., 2020; Yamasaki, 2022). Understanding the neural processes in table tennis, and other open skills sports (Gu et al., 2019), could provide insight into the basis for these neurophysiological benefits.

A few groups explored brain activity during actual table tennis play (Hülsdünker et al., 2020; Carius et al., 2022; Visser et al., 2022). In a recent study, Hülsdünker et al. (2020) compared motion-onset visual evoked potentials (N2) over temporoparietal electrodes for a computer-based and sport-specific stimulus. They found it was feasible to evoke the N2 potential while hitting with a ball machine. However, their study focused on event-related potentials (ERPs), which are neural responses that contains only evoked activity. Any brain activity that is not exactly synchronized in time and phase cancels out (Pfurtscheller, 1992; Van Dijk et al., 1992; Makeig, 1993; Tallon-Baudry et al., 1996). Time frequency analysis of the total (evoked and induced) activity is an alternative way to visualize brain data, often displayed as event-related spectral perturbations (ERSPs; Delorme and Makeig, 2004). The resulting ERSP power fluctuations are time-locked, contain phase-coherent and phase-incoherent neural data, and may reveal aspects of brain dynamics that event-related potentials fail to capture.

Our goal was to determine the electrocortical dynamics of the parieto-occipital cortices during real-world table tennis. We computed power spectral densities (PSDs), event-related spectral perturbations, intertrial phase coherences (ITPCs), event-related potentials, and event-related coherences, as participants played with a human and machine, which gave us a broad neural evaluation of the sport. We hypothesized that parietal cortex would show spectral power fluctuations tied to hit events since various studies show parietal power changes during separate phases of movement planning and execution (Machado et al., 2008; Perfetti et al., 2011; Rawle et al., 2012; Park et al., 2013). We focused our analysis on the parieto-occipital cortices because these regions have been shown to have an important role in real-world interceptive tasks (Senot et al., 2008; Van Der Werf et al., 2010a; Fumuro et al., 2015; Reid and Dessing, 2018; Nordin et al., 2019).

We analyzed human and machine trials separately to account for different anticipatory cues (Hodges et al., 2021), but we did not have a definitive prediction for how the trials would compare. The human player may give better visual cues based on body language, so we may expect stronger event-related potentials and higher magnitude of event-related desynchronization during movement planning (Neuper and Pfurtscheller, 2001). Although, the trajectory and timing of the human’s hits are inherently more variable than the machine, so human trials could arguably be less predictable. If so, then we would expect weaker event-related potentials and lower magnitude of event-related desynchronization during movement planning. This study focused on parietal-occipital cortices, but we also reported results from other brain regions, which provides a comprehensive understanding of the results that could aid in future studies.

## Materials and Methods

### Participants

**Figure 1.**
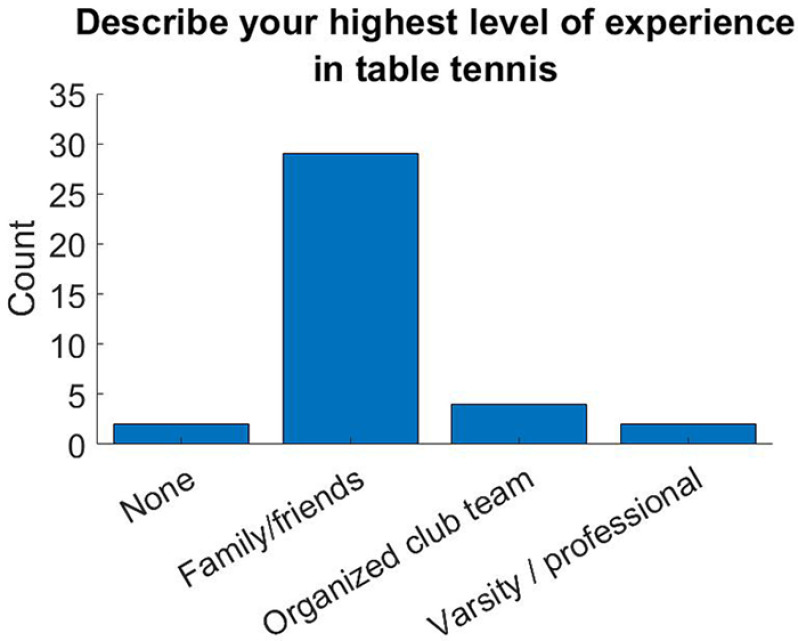
Results from a skill level and experience questionnaire asking participants to describe their highest level of experience in table tennis (multiple choice). For the full skill survey results, refer to Extended Data Figure 1-1.

We recruited 37 participants (ages 23.5 ± 6.7 years, 13 females) with a wide range of skill level and experience. A power analysis revealed that a sample size of 18 participants would be needed to detect a large effect size (Cohen’s *d* = 0.80) with sufficient power (90%) to carry out a two-tailed *t* test comparing ERP response times to different visual motion onset stimuli over temporoparietal electrodes (Hülsdünker et al., 2019). However, we collected more participants because of anticipated data quality issues for our mobile paradigm (e.g., poor contact between the electrodes and scalp, too much artifact) and by following a recent review on neuroimaging guidelines for sample size (Szucs and Ioannidis, 2020). The majority of participants’ experience in table tennis was limited to casual play with family and friends (Fig. 1; Extended Data Fig. 1-1). All participants were right-handed, had normal or corrected-to-normal vision, and self-reported as being fit and able to play. Our exclusion criteria were an inability to follow instructions or make contact with the ball. All participants gave written informed consent, and the University of Florida Institutional Review Board approved the study’s protocol.

10.1523/ENEURO.0463-22.2023.f1-1 Extended Data Figure 1-1 Results from the full survey for gauging participant’s skill level and experience in table tennis and racquet sports. Download Figure 1-1, TIF file.

### Equipment and hardware

Participants wore a dual-layer EEG system (Nordin et al., 2018; Studnicki et al., 2022). The 120 scalp and 120 noise BrainVision ActiCAP snap electrodes logged data on four LiveAmp 64 amplifiers sampled at 500 Hz. Following a dual-layer EEG approach, noise electrodes were mechanically coupled and electrically isolated from scalp electrodes so that each pair of electrodes experienced similar nonphysiological artifacts (i.e., cable movement, electro-magnetic interference, electrode movement). We re-purposed eight of the original 128 scalp electrodes (TP9, P9, PO9, O9, O10, PO10, P10, and TP10) to measure neck muscle activity of the left/right and upper/lower sternocleidomastoid and trapezius. The online reference (CPz) and ground (Fpz) electrodes for the scalp and noise layers were kept separate. Before the start of the experiment, we acquired a 3D head scan of the electrode locations using a Structure Sensor (Occipital Inc.) attached to an iPad with itSeez3D software.

The accelerometer data from inertial measurement units (Cometa WaveTrack Inertial System, 2000 Hz) on the handles of two wooden paddles and on the ball machine (Robo-Pong 2040+) allowed us to compute the timing of hit events. We also placed inertial measurement units on the table tennis table, net, participant’s backpack, forehead, and lower back for a subset of participants for a different analysis. Each sensor logged the data independently and was synchronized with the EEG system using pulses from an Arduino. Following an approach from Artoni and colleagues, a TTL pulse was sent to the BrainVision EEG system at the same time as an analog pulse was sent to a Cometas sensor (Artoni et al., 2018).

We recorded videos (GoPro Hero 7, range of 30–240 fps) for all trials to inspect the accuracy of the hit events. Hit events were manually marked in the videos using Adobe Premiere Pro software and exported as hit timing relative to the first hit event. We then aligned the relative hit events to the resultant acceleration of the paddle IMU data using cross-correlation (xcorr function in MATLAB). We removed any inertial measurement unit events outside of 200 ms from the video markers to filter out mislabeled hits (e.g., if the participant hits the table with their paddle by accident).

### Experimental protocol

**Figure 2.**
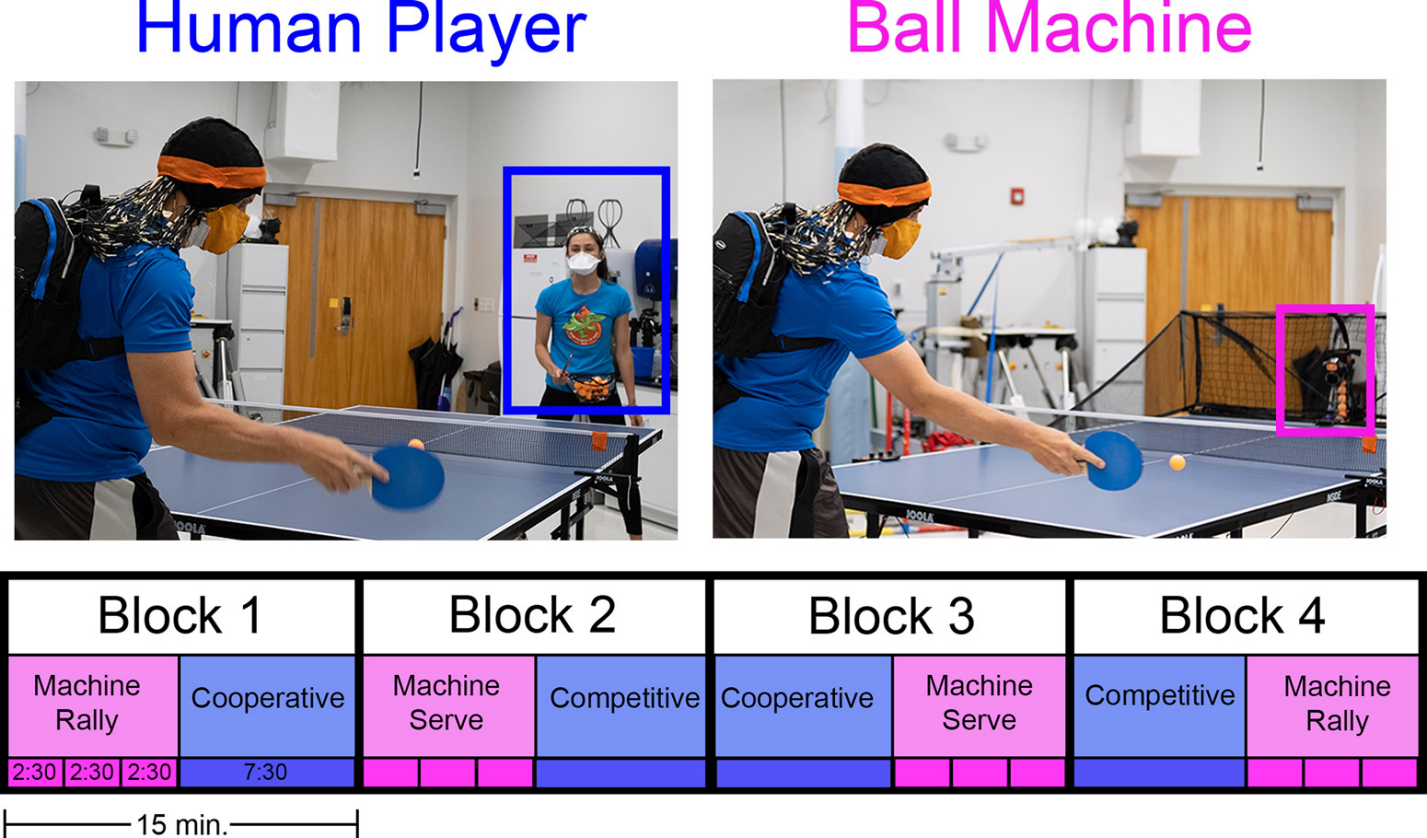
Table tennis experimental protocol. Participants played for four blocks (each 15 min long). Within each block, participants played 7.5 min with a human player (blue) and 7.5 min with a ball machine (pink). Human player trials were cooperative or competitive. Ball machine trials replicated a rally shot (1 bounce) or serve (two bounces) and alternated between moving and stationary conditions (each 2.5 min). The order of trials within blocks was randomized. A video of the different table tennis conditions can be seen in Movie 1.

Participants played table tennis in four blocks of 15 min (Fig. 2). In a single block, participants played one trial with a human player (cooperatively or competitively) for 7.5 min and played with a ball machine for 2.5 min in three back-to-back trials. The ball machine trials alternated between stationary and moving trials. For the stationary trials, the ball’s trajectory was predictable, and the participants did not have to move their feet to hit the ball. For the moving trials, the ball machine oscillated and fed balls toward all directions on the table. Each block of trials with the ball machine had either one or two bounces on the table before the participant’s hit, simulating a hit or serve, respectively (Movie 1). The ball was fed at a rate of approximately once every 2 s (0.5 Hz), and the angle and speed of the feed were adjusted so that the ball landed halfway between the net and end of the table.

Movie 1. Table tennis conditions. Each block of trials with the ball machine had either one or two bounces on the table before the participant’s hit, simulating a hit or serve, respectively. 10.1523/ENEURO.0463-22.2023.video.1

For cooperative and competitive trials, the human player was experienced enough to scale their play to match the level of each participant. We instructed participants to work together with the human player to keep the ball in play during cooperative trials. For competitive trials, participants were instructed to try winning a 21-point game against the human player, switching serves every five points. The order of trials among and within blocks was randomized. We collected a 5-min standing baseline at the beginning and end of all the table tennis trials. The total amount of data collected, excluding breaks, was 70 min.

### MRI acquisition

On a different day, we collected a T1 structural MR image of the participant’s head using a 3T Philips Ingenia Scanner. The sequence was a magnetization-prepared rapid-acquisition gradient-echo (MP-RAGE) with a 32-channel head coil. Sixteen of the participants were scanned with the following parameters: 7.00 ms repetition time (TR), 3.17 ms echo time (TE), 8° flip angle, 240 mm × 240 mm × 176 mm field of view, and 1 mm^3^ recon voxel size. The remaining participants were scanned with the following parameters: 11.13 ms repetition time (TR), 5.10 ms echo time (TE), 8° flip angle, 256 × 240 × 179.9 mm field of view, and 0.67 × 0.67 × 0.70 mm^3^ recon voxel size.

### EEG preprocessing

**Figure 3.**
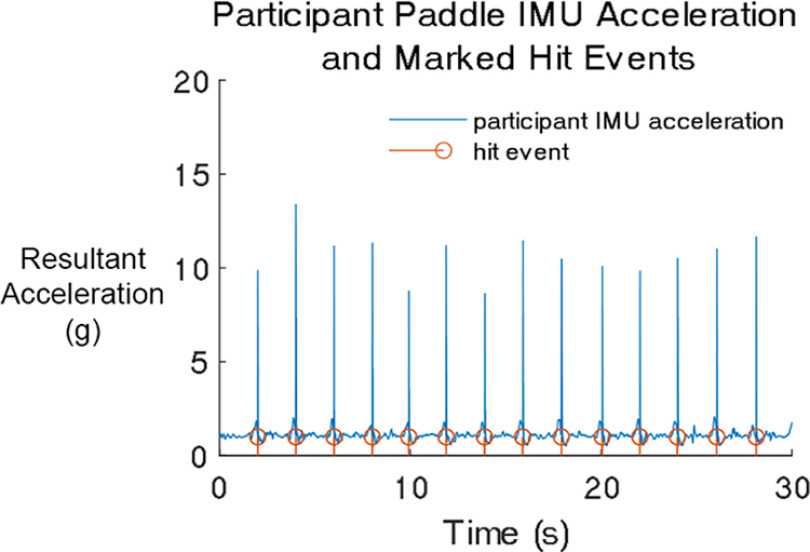
Exemplary inertial measurement unit (IMU) data from a single participant. The blue trace shows the resultant acceleration of the IMU on the participant’s paddle. The orange stem markers show the marked hit events. For hit events from the ball machine, human player paddle, and participant paddle in ball machine and human player conditions, refer to Extended Data Figure 3-1.

We processed data using custom MATLAB R2020A (MathWorks) scripts and open-source packages including EEGLAB (v2021.0), FieldTrip, and a Computational Anatomy Toolbox for SPM (CAT12) functions. Data were 1 Hz high-pass filtered using the eegfiltnew function to remove drift. Scalp, noise, muscle, and IMU data were aligned and merged into a single dataset using the synchronization pulses from the Arduino timer module. We marked hit events when the first derivative of the resultant acceleration of the ball machine or paddle IMUs exceeded 0.75 gravity (Fig. 3; Extended Data Fig. 3-1). We visually inspected and manually separated trials into individual datasets based on continuous blocks of hitting events. For each trial, we used Cleanline to remove 60-Hz line noise (Mullen, 2012), down-sampled the data to 250 Hz, and rejected bad channels that were outside of 3 standard deviations (SDs) away from the median of all other channels. We averaged re-referenced the data with full rank and spherically interpolated the rejected channels. Channel rejection, average re-referencing, and interpolation were performed on scalp and noise electrodes separately.

10.1523/ENEURO.0463-22.2023.f3-1 Extended Data Figure 3-1 Exemplary IMU data from a single participant. The blue traces show the resultant acceleration of the IMUs. The orange stem markers show the marked hit events (either ball machine feeds, human player hits, or participant hits). The yellow markers show the serve hit events. The acceleration data was sufficiently clean to extract the timing of hit events. There were some errors in event marking (like in the ball machine IMU data ∼7 s), but these errors were uncommon. Download Figure 3-1, TIF file.

We used iCanClean to remove motion and muscle artifacts (Downey and Ferris, 2022; Studnicki et al., 2022; Gonsisko et al., 2023). For motion artifact removal, the algorithm used canonical correlation analysis in a 2-s sliding window to find subspaces of the scalp electrodes that were strongly correlated with subspaces of the noise electrodes. The subspaces with the strongest correlation (*r*^2^ > 0.85) were rejected. For muscle artifact removal, we used the same approach but correlated scalp electrodes to the muscle channels and rejected subspaces with *r*^2^ > 0.40. Then, the clean_artifacts function rejected noisy time windows (SD threshold = 30 and window criterion = 0.3).

### Independent component analysis

We ran independent component analysis (ICA) using adaptive mixture independent component analysis (AMICA; Palmer et al., 2006, 2008). Before independent component analysis, we used principal component analysis to ensure rank by reducing the number of principal components by the maximum number of scalp channels that were interpolated across trials for a given participant. The resulting weight matrix was applied to the preprocessed dataset before time window rejection.

### Custom volume conduction model

Individual volume conduction models using the finite element method were created for each participant using the FieldTrip-SimBio pipeline (Vorwerk et al., 2018). We manually marked electrode locations and fiducials (nasion and left/right preauricular points) with EEGLAB get_chanlocs. An inward shift of 10 mm accounted for the dual-layer electrode height from the electrode surface to the scalp. Next, we resliced and segmented the individual MR images into five tissue layers (skin, skull, cerebral spinal fluid, gray matter, and white matter) using the CAT12 plug-in for SPM. Tissue conductivity values were chosen according to (Vorwerk et al., 2019; skin: 430, skull: 10, cerebral spinal fluid: 1790, gray matter: 330, white matter: 140 mS/m). We meshed the tissues into hexahedrons (0.3 shift and 1 mm resolution), created the head model using the SimBio finite element method, and aligned the head model with the electrode locations using fiducials. Finally, we computed the source model, transfer matrix, and leadfield matrix using ft_prepare_sourcemodel, ft_prepare_vol_sens, and ft_prepare_leadfield, respectively.

### Source localization

We fit each independent component to an equivalent dipole with DIPFIT3.3 using our custom head models, converted the dipole locations to Montreal Neurological Institute (MNI) space, and retained components that explained > 85% of the scalp variance (Oostenveld and Oostendorp, 2002). We removed components that were labeled most popularly (Klug and Gramann, 2021) as nonbrain sources by the ICLabel algorithm (Pion-Tonachini et al., 2019), and further removed nonbrain components based on visual inspection of the dipole location and power spectra. Any participants that contributed less than five brain components were removed from subsequent analyses [25 participants remained, with median (IQR) 9 (8) components per participant]. The remaining brain components were clustered by dipole location using k-means. We applied the silhouette algorithm (Rousseeuw, 1987) to obtain the optimum number of clusters (k = 10). Dipoles > 3 SDs from the final clusters were assigned as outliers. To avoid artificially inflating sample sizes, we retained a single component per participant in each cluster that explained the greatest amount of scalp variance (i.e., whichever component that best fit the single dipole model from DIPFIT3.3). On average, nine components were removed per cluster.

### Human versus machine

To account for any effects in total power differences between ball machine and human player trials, we calculated the average EEG log power spectral density (PSD). Individual power spectral densities were computed with the spectopo function (pwelch method with nonoverlapping hamming windows, 250-window length). We used the FOOOF toolbox (Donoghue et al., 2020) to separate the aperiodic and periodic components by modeling each power spectra from 3 to 40 Hz (peak width limits: [1 8], minimum peak height: 0.05, and maximum number of peaks: 2). We computed a flattened PSD by removing the aperiodic component from the original PSD. To test for significance between ball machine and human player power spectral densities, we performed a two-sided Wilcoxon signed rank test across all participants at each frequency on the flattened power spectra. To account for type 1 error, we adjusted our results using the false discovery rate correction with the Benjamini and Hochberg method (Benjamini and Hochberg, 1995).

We assessed electrocortical activity tied to hit events in an average “swing cycle” using event-related spectral perturbations (ERSPs). We defined the events of a “swing cycle” as: (1) when the ball appeared to the participant (as a feed from the ball machine or as a hit from the human player); (2) when the ball made contact with the participant’s paddle; and (3) when the next ball appeared to the participant. We epoched the data 1.0–2.5 s around the time when the ball appeared to the participant and rejected 10% of epochs that had the highest voltage maxima, resulting in (mean ± SD) 546 ± 144 epochs with the ball machine and 543 ± 111 epochs with the human player. Single trial spectrograms were calculated with newtimef (Morlet wavelets whose cycles increased linearly with frequency from 3 to 64 cycles). We took the mean event-related spectral perturbation across components in each parieto-occipital cluster to create a grand mean ERSP. Each ERSP was linearly time warped to the median latencies of the hit events across all ball machine and human player trials. The spectral baseline was the average power across the swing cycle for all conditions. To find statistically significant time-frequency changes within each condition, we bootstrapped individual event-related spectral perturbations (0.05 α, shuffled along the time dimension, 2000 iterations). We corrected for multiple comparisons using false discovery correction with the Benjamini and Hochberg method (Benjamini and Hochberg, 1995), and nonsignificant values were set to zero. To assess differences across human player and ball machine conditions, we used nonparametric permutation statistics with cluster-based multiple comparison correction (0.05 α, 2000 iterations; M.X. Cohen, 2014).

Event-related potentials (ERPs) and intertrial phase coherence (ITPC) measured the consistency of phase across trials. For both the ERP and ITPC calculation, we epoched and extracted time-frequency data for each parietal component like for the ERSP, but without time warping. The event-related potential was averaged across participants. We took the mean intertrial phase coherence across components in each parieto-occipital cluster to create a grand mean ITPC, bootstrapped the individual ITPCs, and masked for significance at an α of 0.05.

We assessed the functional connectivity between parieto-occipital clusters using event-related phase coherence (Delorme and Makeig, 2004). Only the participants who contributed a component to both clusters for each pairwise cluster comparison were chosen (mean ± SD = 14 ± 2 participants). We then set the epoch latency limits −0.5–1.0 s around the time when the ball appeared to the participant and used the function newcrossf to compute the event-related coherence between clusters for each participant. Similar to the ERSP calculation, we took the mean event-related coherence across participants for each comparison. To find statistically significant time-frequency coherence, we bootstrapped the individual event-related coherence spectrograms, and masked for significance at an α of 0.05 (shuffled along the time dimension, 2000 iterations). To assess differences across human and machine conditions, we used nonparametric permutation statistics with cluster-based multiple comparison correction (0.05 α, 2000 iterations).

### Rally versus serve

We further probed the differences between ball machine and human trial conditions by separating the participant’s hits after receiving the ball during a rally or after a serve. For trials with the human player, participants received the ball on one bounce during a rally. Participants received the ball on two bounces while receiving a serve from the human player. For trials with the ball machine, we adjusted the angle of the ball machine to replicate an incoming shot on either one or two bounces. Five participants did not have rally data with the ball machine, so we excluded these participants from the analysis. There were (mean ± SD) 287 ± 40 epochs for ball machine rallies, 274 ± 51 epochs for ball machine serves, 472 ± 112 epochs for human rallies, and 67 ± 16 for human serves. Since there were fewer epochs with human serves, we randomly sampled 70 epochs per participant per condition so that the number of epochs were similar across conditions. We then repeated our analyses of ERSPs, ERPs, and ITPCs for the rally and serve trials for each of the parieto-occipital clusters. We did not repeat the event-related coherence analysis for brevity.

### Data and code availability

Files for parieto-occipital component data, organized in MATLAB structures (63.5 MB) can be accessed at doi: 10.17 632/gjr7pjn2m8.1. Each parieto-occipital cluster data file contains independent components (IC_##) and their MNI position coordinates (mnipos), component activations (icaact), and time-warp latencies for the ERSP plots. Data were epoched around receiving the ball (as a hit from the human player or as a feed from the ball machine) for each condition (at time 0). We separated the down-sampled epochs pertaining to rallies with the ball machine, rallies with the human player, serves with the ball machine, and serves with the human player. Data were sampled at 250 Hz and processed as described in the paper. The code used for preprocessing and analyses is available on reasonable request.

## Results

**Figure 4.**
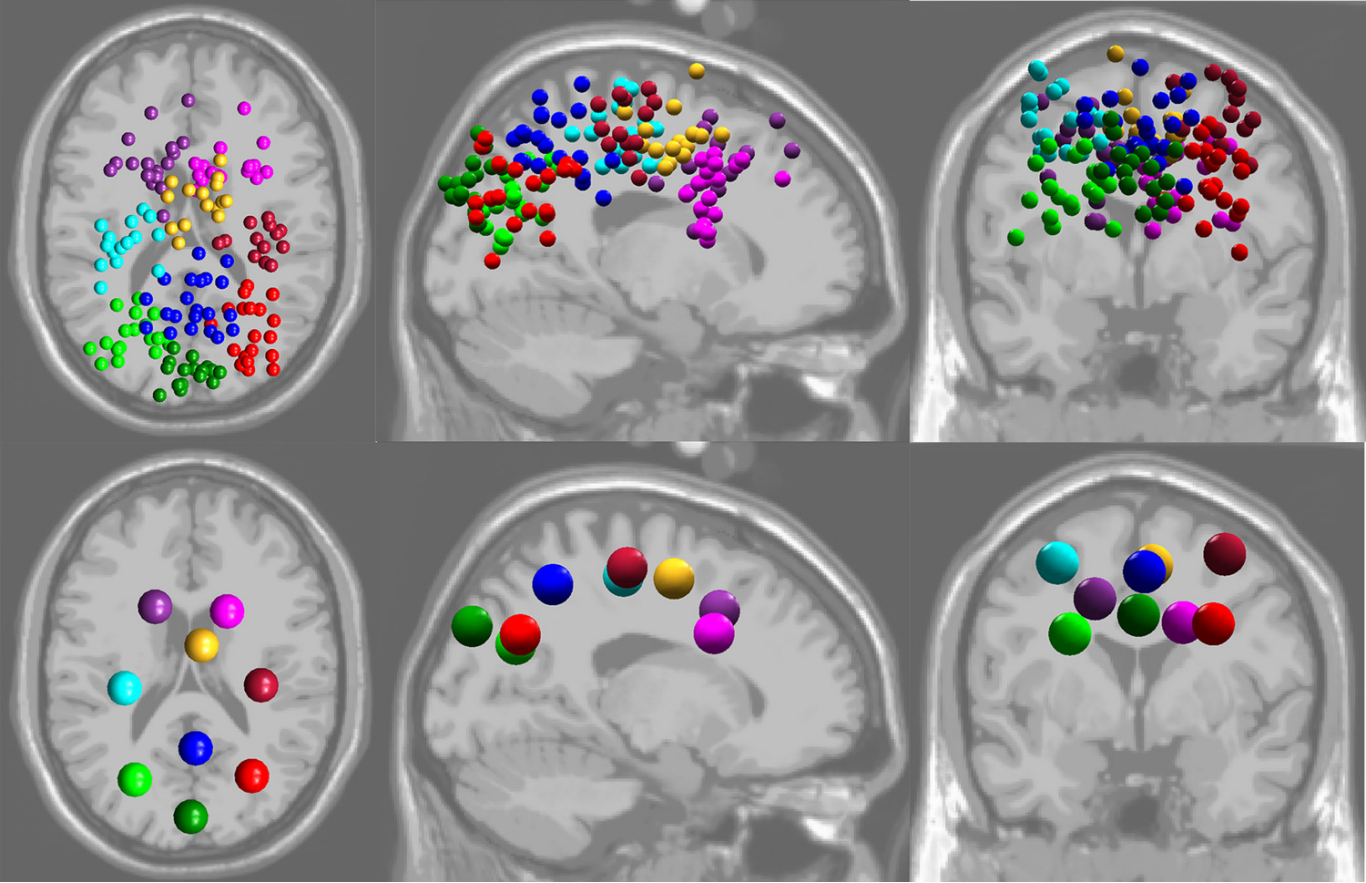
Resulting cortical dipoles shown for all participants (top). Cluster centroids (bottom) are shown in axial (left), sagittal (middle), and coronal (right) planes. We found clusters located in left frontal (purple), right frontal (magenta), left sensorimotor (cyan), supplementary motor area (yellow), right sensorimotor (dark red), left parieto-occipital (light green), precuneus (blue), right parieto-occipital (red), and cuneus (dark green).

The resulting clusters that contained dipoles from more than half of the participants (>13 participants) were left frontal (*n* = 18), right frontal (*n* = 18), left sensorimotor (*n* = 17), supplementary motor area (*n* = 15), right sensorimotor (*n* = 13), left parieto-occipital (*n* = 18), precuneus (*n* = 21), right parieto-occipital (*n* = 19), and cuneus (*n* = 16). Figure 4 shows the dipole locations plotted on the standard MNI template. However, for this analysis, we focused on the parieto-occipital area (left and right parieto-occipital cortex, precuneus, and cuneus). Table 1 shows the MNI and Talairach coordinates of the parieto-occipital cluster centroids.

**Table 1:** Coordinates of the parieto-occipital cluster centroids in Talairach and Montreal Neurological Institute (MNI) space

Cluster	Talairach coordinates	MNI coordinates
Left parieto-occipital	−26 −65 28	−26.0 −68.2 26.7
Right parieto-occipital	29 −63 31	29.3 −66.5 30.5
Precuneus	3 −49 49	2.7 −53.4 50.6
Cuneus	0 −82 35	0.5 −85.8 33.9

### Human versus machine

**Figure 5.**
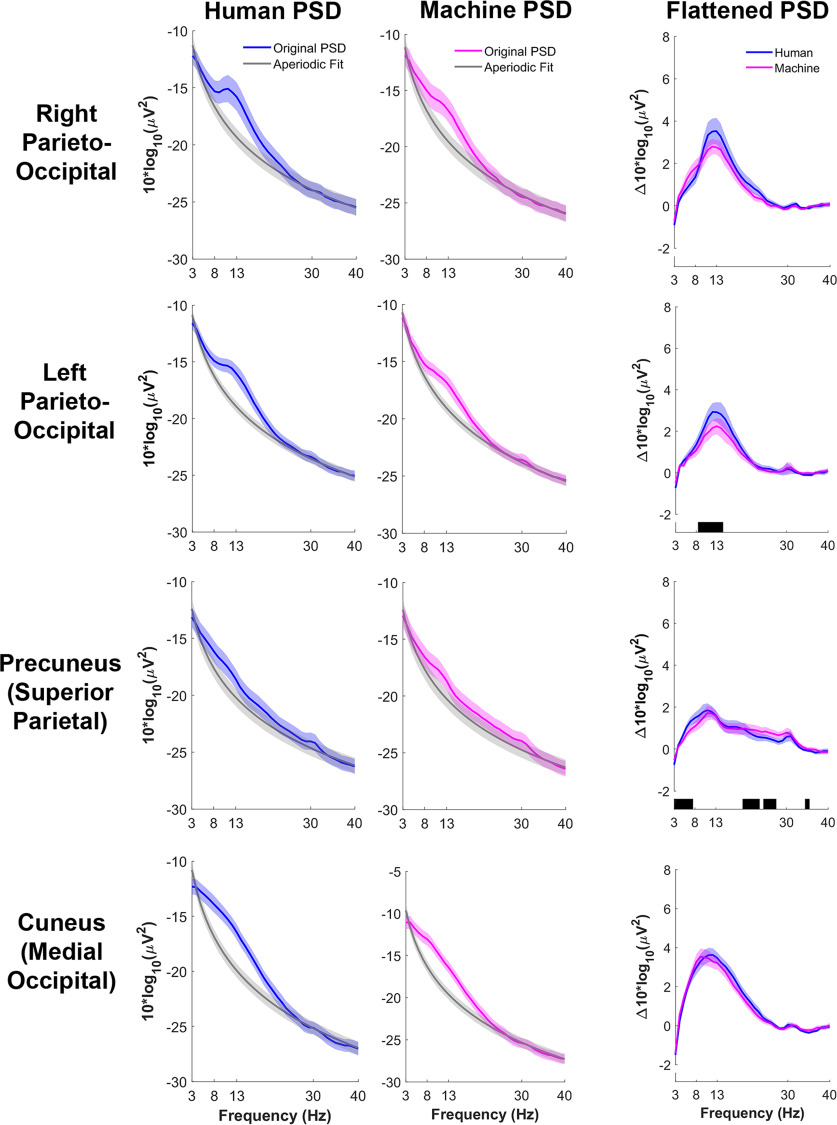
Average ± standard error (SE) power spectra for human (left, blue) and ball machine (middle, magenta) trials. Average +/– SE power spectral density (PSD) plots aperiodic fit from the FOOOF model is in gray. The flattened spectra (right) of both human (blue) and machine (magenta) trials was calculated by taking the difference between the original power spectra and the aperiodic component. The black shading along the *x*-axis of the flattened spectra reflects significant differences after multiple comparisons correction between human and machine conditions (*p* < 0.05).

Power spectral density (PSD) plots of the parieto-occipital clusters all contained a nonoscillatory component (modeled by the aperiodic fit curve) and an oscillatory component (the flattened PSD in Fig. 5). Oscillatory power for human and machine trials was primarily located in the α and low β bands for all parieto-occipital clusters. Human trials had significantly greater α power in left parieto-occipital cortex and precuneus than machine trials (*p* < 0.05). Machine trials had significantly greater β power in precuneus than human trials. There were no significant differences in power between human and machine conditions for right parieto-occipital cortex and cuneus.

All parieto-occipital clusters had event-related spectral perturbation (ERSP) plots with significant spectral power fluctuations in θ, α, and β bands tied to hit events. The intertrial phase coherence (ITPC) and event-related potentials revealed phase-locked activity between receiving and hitting the ball for ball machine trials only.

**Figure 6.**
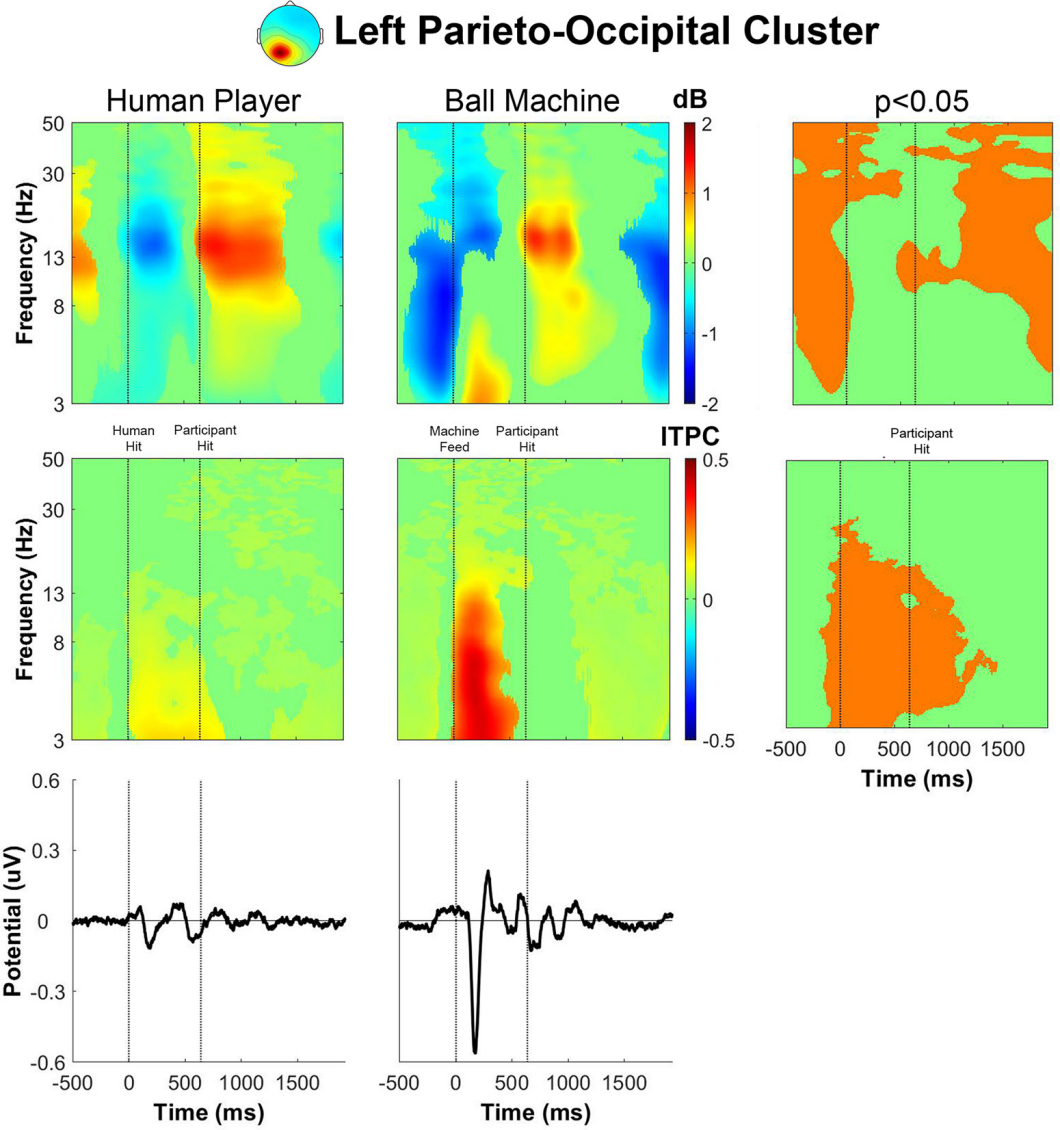
Left parieto-occipital cluster group average (*n* = 18) results of human player trials (left) and ball machine trials (middle). Significant differences between human player and ball machine trials (right) are in orange (*p* < 0.05). Top row, Event-related spectral perturbation (ERSP) plots. Significant increases in spectral power within condition relative to baseline are in red and significant decreases in spectral power relative to baseline are in blue (*p* < 0.05). Single trial spectrograms were warped to the event timing of the average swing cycle. At time 0, the ball appeared (either the human player made contact or the ball machine fed the ball). At 640 ms, the participant hit the ball. At 1924 ms, the next ball appeared. Spectral baseline was the average power across the entire epoch in both conditions. Middle row, Intertrial phase coherence (ITPC) plots, event-locked to when the ball appeared (time 0). Significant increases in intertrial phase coherence are in red (*p* < 0.05). Bottom row, Mean event-related potential (ERP) plots, event-locked to when the ball appeared (time 0). Intertrial phase coherence and event-related potentials were not warped to the hit events, but the same average event timings are overlaid.

The event-related spectral perturbation for human player trials in the left parieto-occipital cluster (Fig. 6) had event-related desynchronization in α and β power after receiving the ball, followed by event-related synchronization right before the participant hit the ball. The event-related spectral perturbation for ball machine trials exhibited some differences from the human player trials. There was additional θ and α desynchronization before receiving the ball with θ synchronization ∼200 ms before hitting the ball. The intertrial phase coherence for the left parieto-occipital cluster increased in θ and α bands for ∼500 ms after the ball fed from the ball machine. A large deflection in the event-related potential occurred at a similar time as the increase in intertrial phase coherence for the ball machine trials. There was much less phase-locked activity in the human trials than in the ball machine trials.

**Figure 7.**
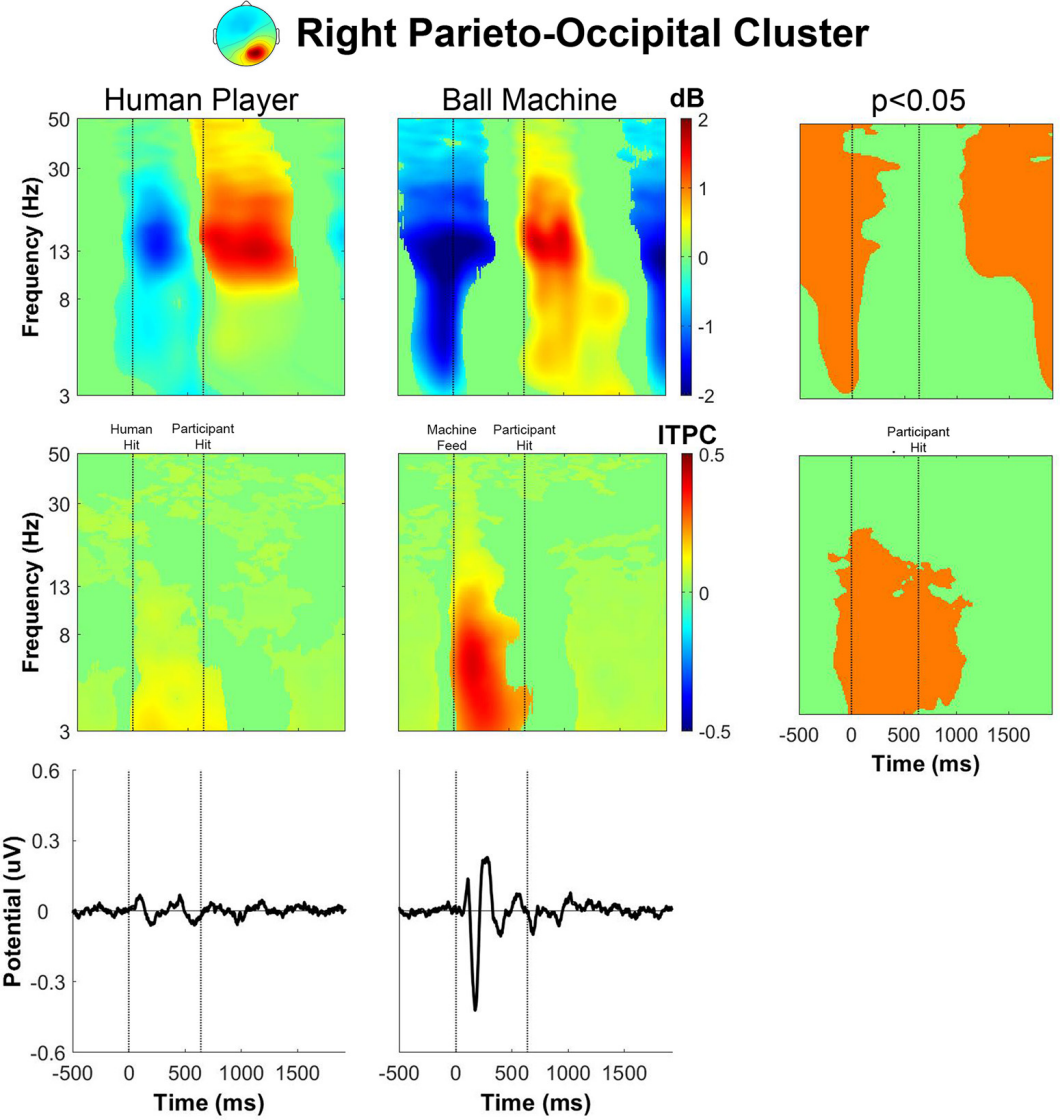
Right parieto-occipital cluster group average (*n* = 19) results of human player trials (left) and ball machine trials (middle). Significant differences between human player and ball machine trials (right) are in orange (*p* < 0.05). Top row, Event-related spectral perturbation (ERSP) plots. Significant increases in spectral power within condition relative to baseline are in red and significant decreases in spectral power relative to baseline are in blue (*p* < 0.05). Single trial spectrograms were warped to the event timing of the average swing cycle. At time 0, the ball appeared (either the human player made contact or the ball machine fed the ball). At 640 ms, the participant hit the ball. At 1924 ms, the next ball appeared. Spectral baseline was the average power across the entire epoch in both conditions. Middle row, Intertrial phase coherence (ITPC) plots, event-locked to when the ball appeared (time 0). Significant increases in intertrial phase coherence are in red (*p* < 0.05). Bottom row, Mean event-related potential (ERP) plots, event-locked to when the ball appeared (time 0). Intertrial phase coherence and event-related potentials were not warped to the hit events, but the same average event timings are overlaid.

The pattern of α and β spectral power fluctuations for the human trials in the right parieto-occipital cluster (Fig. 7) were very similar to the pattern of spectral power fluctuations of the left parieto-occipital cluster from Figure 6. For the ball machine trials, the event-related spectral perturbation showed a broadband decrease in θ, α, and β power immediately before receiving the ball, a decrease in α and β power immediately after receiving the ball, and a broadband increase in θ, α, and β power immediately after the participant hit the ball. There was no significant θ synchronization in the ball machine trials between feed and hit events in the right parieto-occipital cluster as we saw in the left parieto-occipital cluster. The intertrial phase coherence for the right parieto-occipital cluster increased for θ and α bands between feed and hit events for ball machine trials. A large deflection in the event-related potential occurred around 200 ms after the ball machine fed the ball. Similar to what we observed in the left parieto-occipital cluster, there was much less phase-locked activity in the human trials than in the ball machine trials for the right parieto-occipital cluster.

**Figure 8.**
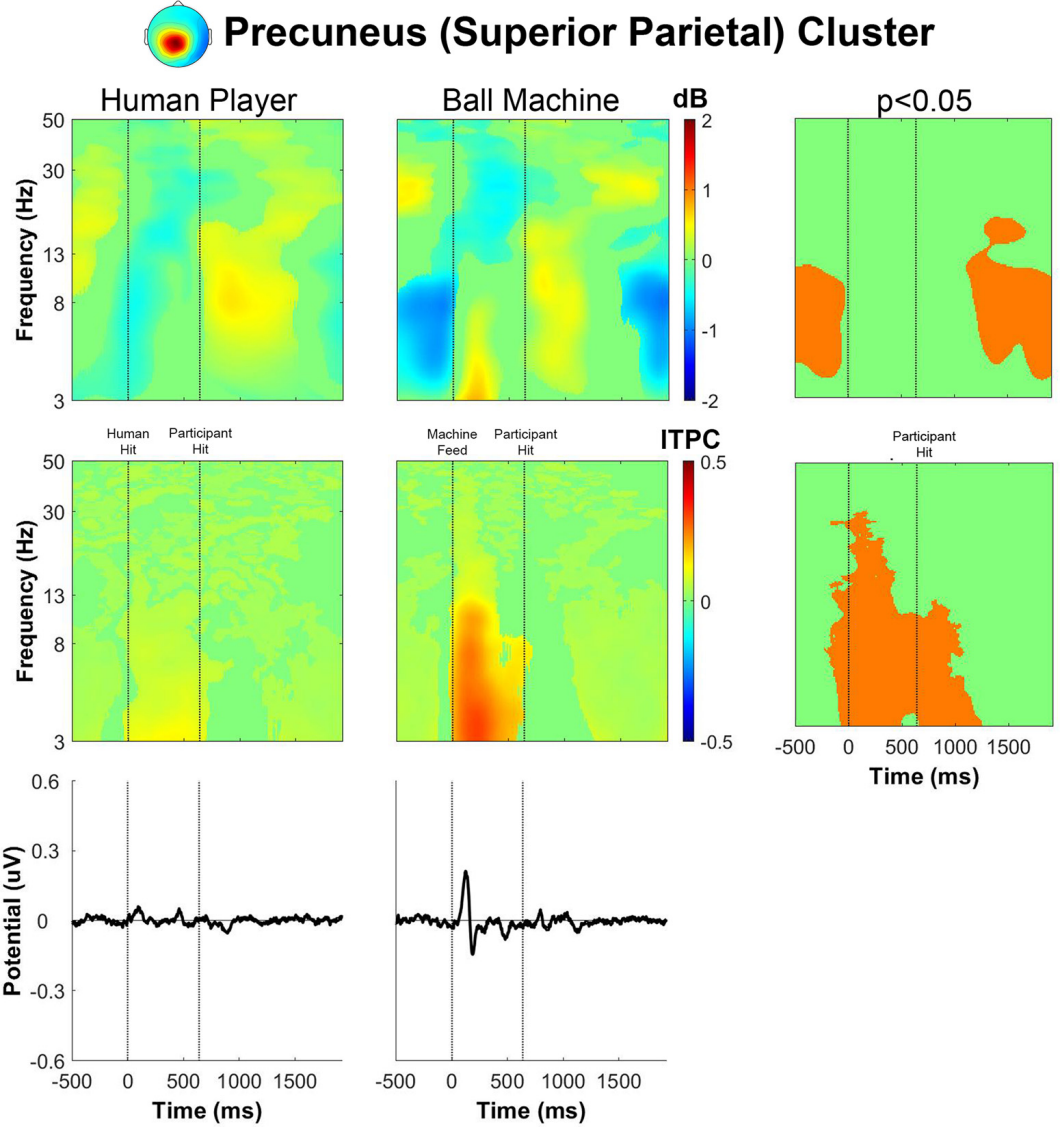
Precuneus (superior parietal) cluster group average (*n* = 21) results of human player trials (left) and ball machine trials (middle). Significant differences between human player and ball machine trials (right) are in orange (*p* < 0.05). Top row, Event-related spectral perturbation (ERSP) plots. Significant increases in spectral power within condition relative to baseline are in red and significant decreases in spectral power relative to baseline are in blue (*p* < 0.05). Single trial spectrograms were warped to the event timing of the average swing cycle. At time 0, the ball appeared (either the human player made contact or the ball machine fed the ball). At 640 ms, the participant hit the ball. At 1924 ms, the next ball appeared. Spectral baseline was the average power across the entire epoch in both conditions. Middle row, Intertrial phase coherence (ITPC) plots, event-locked to when the ball appeared (time 0). Significant increases in intertrial phase coherence are in red (*p* < 0.05). Bottom row, Mean event-related potential (ERP) plots, event-locked to when the ball appeared (time 0). Intertrial phase coherence and event-related potentials were not warped to the hit events, but the same average event timings are overlaid.

The magnitude of power fluctuations in the precuneus (superior parietal) cluster (Fig. 8) from the spectral baseline was smaller than the other clusters, but the pattern of activity was similar, but not identical, to the left parieto-occipital cluster. The event-related spectral perturbation for human player trials had a significant decrease in θ and α power immediately before receiving the ball. There was also a decrease in α and β power immediately after receiving the ball and an increase in α power immediately after the participant hit the ball. For the ball machine trials, θ desynchronization before receiving the ball was followed by θ synchronization 200 ms before hitting the ball, similar to the pattern we observed in the left parieto-occipital cluster. The intertrial phase coherence for the precuneus cluster had a higher increase for θ and α bands after receiving the ball in ball machine trials than in human trials. Like in the other clusters, there was a noticeable event-related potential in the ball machine trials that was absent in the human trials.

**Figure 9.**
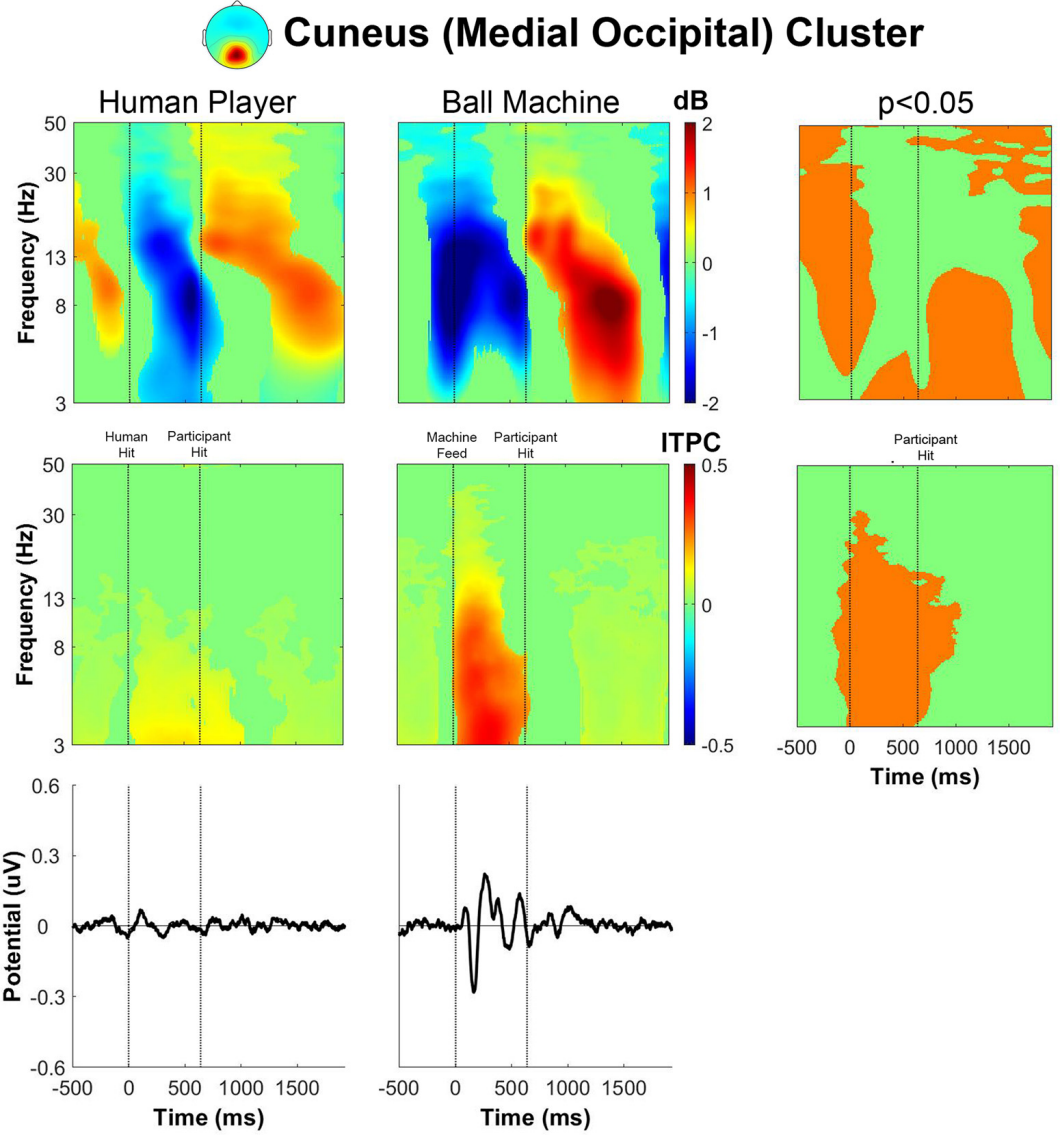
Cuneus (medial occipital) cluster group average (*n* = 16) results of human player trials (left) and ball machine trials (middle). Significant differences between human player and ball machine trials (right) are in orange (*p* < 0.05). Top row, Event-related spectral perturbation (ERSP) plots. Significant increases in spectral power within condition relative to baseline are in red and significant decreases in spectral power relative to baseline are in blue (*p* < 0.05). Single trial spectrograms were warped to the event timing of the average swing cycle. At time 0, the ball appeared (either the human player made contact or the ball machine fed the ball). At 640 ms, the participant hit the ball. At 1924 ms, the next ball appeared. Spectral baseline was the average power across the entire epoch in both conditions. Middle row, Intertrial phase coherence (ITPC) plots, event-locked to when the ball appeared (time 0). Significant increases in intertrial phase coherence are in red (*p* < 0.05). Bottom row, Mean event-related potential (ERP) plots, event-locked to when the ball appeared (time 0). Intertrial phase coherence and event-related potentials were not warped to the hit events, but the same average event timings are overlaid. Refer to the extended data for other cluster group average results: right frontal (Extended Data Fig. 9-1), left frontal (Extended Data Fig. 9-2), supplementary motor area (Extended Data Fig. 9-3), right sensorimotor (Extended Data Fig. 9-4), and left sensorimotor (Extended Data Fig. 9-5).

The event-related spectral perturbation for human player trials in the cuneus (medial occipital) cluster (Fig. 9) had broadband θ, α, and β desynchronization after receiving the ball and before the participant hit the ball. There was also β synchronization immediately after the participant made contact with the ball, followed by θ and α synchronization before receiving the next ball. For the ball machine trials, the spectral power fluctuations appeared to shift earlier than in the human trials. The event-related spectral perturbations showed a broadband decrease in θ, α, and β power preceding the feed from the ball machine, a decrease in α and β power after receiving the feed, and an increase in θ, α, and β power after the participant hit the ball. The intertrial phase coherence for the cuneus cluster was markedly similar to the other clusters, which showed an increase in θ and α phase coherence after receiving the ball in ball machine trials only. A deflection in the event-related potential occurred at a similar time as the increase in intertrial phase coherence for ball machine trials only.

10.1523/ENEURO.0463-22.2023.f9-1 Extended Data Figure 9-1 Right frontal cluster group average (*n* = 18) results. Download Figure 9-1, TIF file.

10.1523/ENEURO.0463-22.2023.f9-2 Extended Data Figure 9-2 Left frontal cluster group average (*n* = 18) results. Download Figure 9-2, TIF file.

10.1523/ENEURO.0463-22.2023.f9-3 Extended Data Figure 9-3 Supplementary motor area cluster group average (*n* = 15) results. Download Figure 9-3, TIF file.

10.1523/ENEURO.0463-22.2023.f9-4 Extended Data Figure 9-4 Right sensorimotor cluster group average (*n* = 13) results. Download Figure 9-4, TIF file.

10.1523/ENEURO.0463-22.2023.f9-5 Extended Data Figure 9-5 Left sensorimotor cluster group average (*n* = 17) results. Download Figure 9-5, TIF file.

We found a similar pattern of event-related activity in the right frontal (Extended Data Fig. 9-1), left frontal (Extended Data Fig. 9-2), supplementary motor area (Extended Data Fig. 9-3), right sensorimotor (Extended Data Fig. 9-4), and left sensorimotor (Extended Data Fig. 9-5) clusters. All of these clusters showed an increase in θ and α intertrial phase coherence after receiving the ball in the ball machine trials whereas the human player trials did not. The right frontal, supplementary motor area, and left sensorimotor clusters showed significant differences in spectral power fluctuations between human player and ball machine trials.

**Figure 10.**
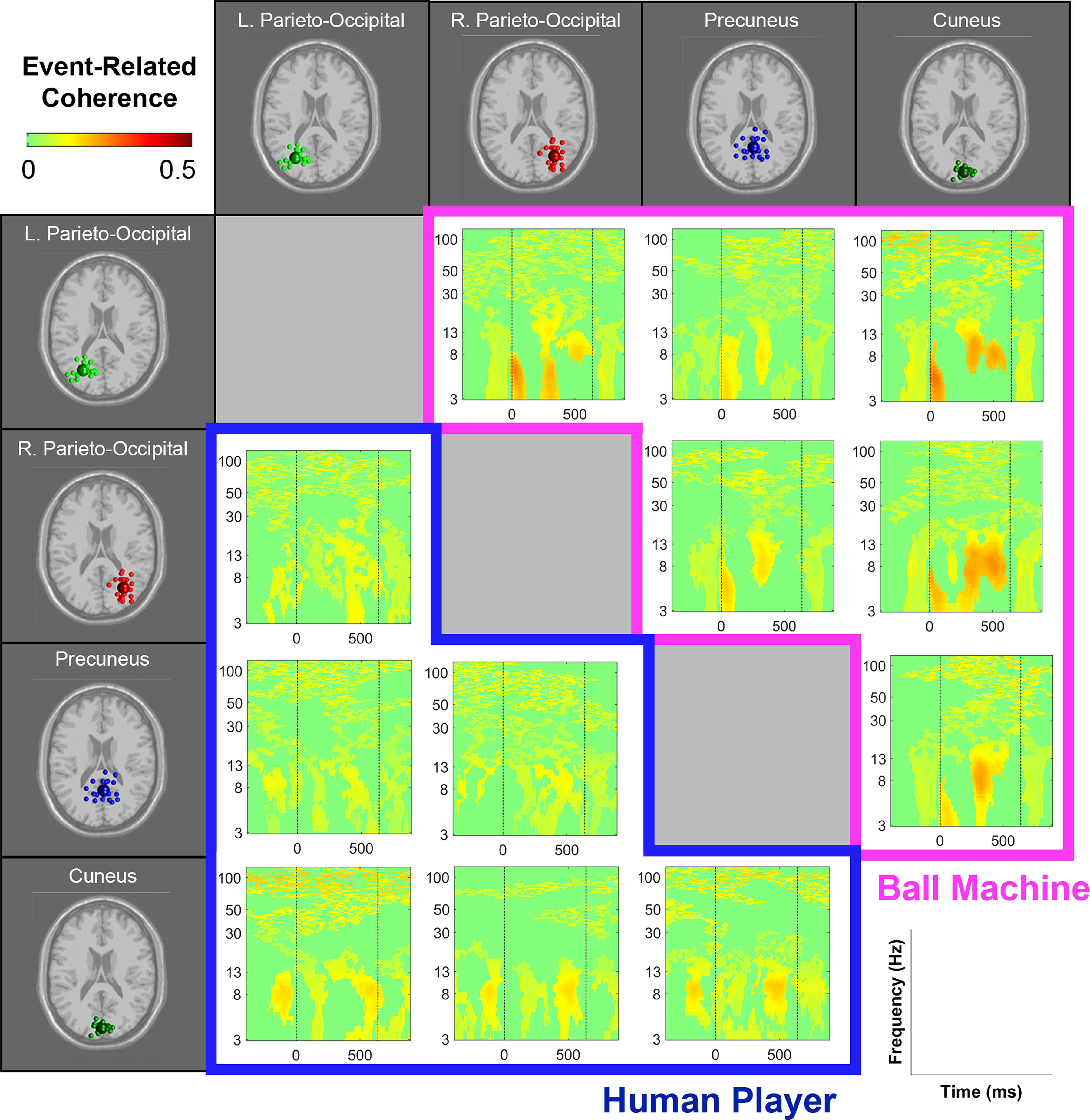
Event-related coherence between parieto-occipital clusters during human player trials (blue, bottom diagonal) and ball machine trials (magenta, top diagonal). Significant increases in event-related coherence are in red (*p* < 0.05). At time 0, the ball appeared (either the human player made contact or the ball machine fed the ball). The data were not time-warped, but the average time of the participant hit across trials was overlaid (time 640 ms). Only the participants who contributed a component to both clusters for each pairwise cluster comparison were chosen (15 participants: left parieto-occipital × right-parieto-occipital; 15 participants: left parieto-occipital × precuneus; 12 participants: left parieto-occipital × cuneus; 16 participants: right parieto-occipital × precuneus; 12 participants: right parieto-occipital × cuneus; 15 participants: precuneus × cuneus).

**Figure 11.**
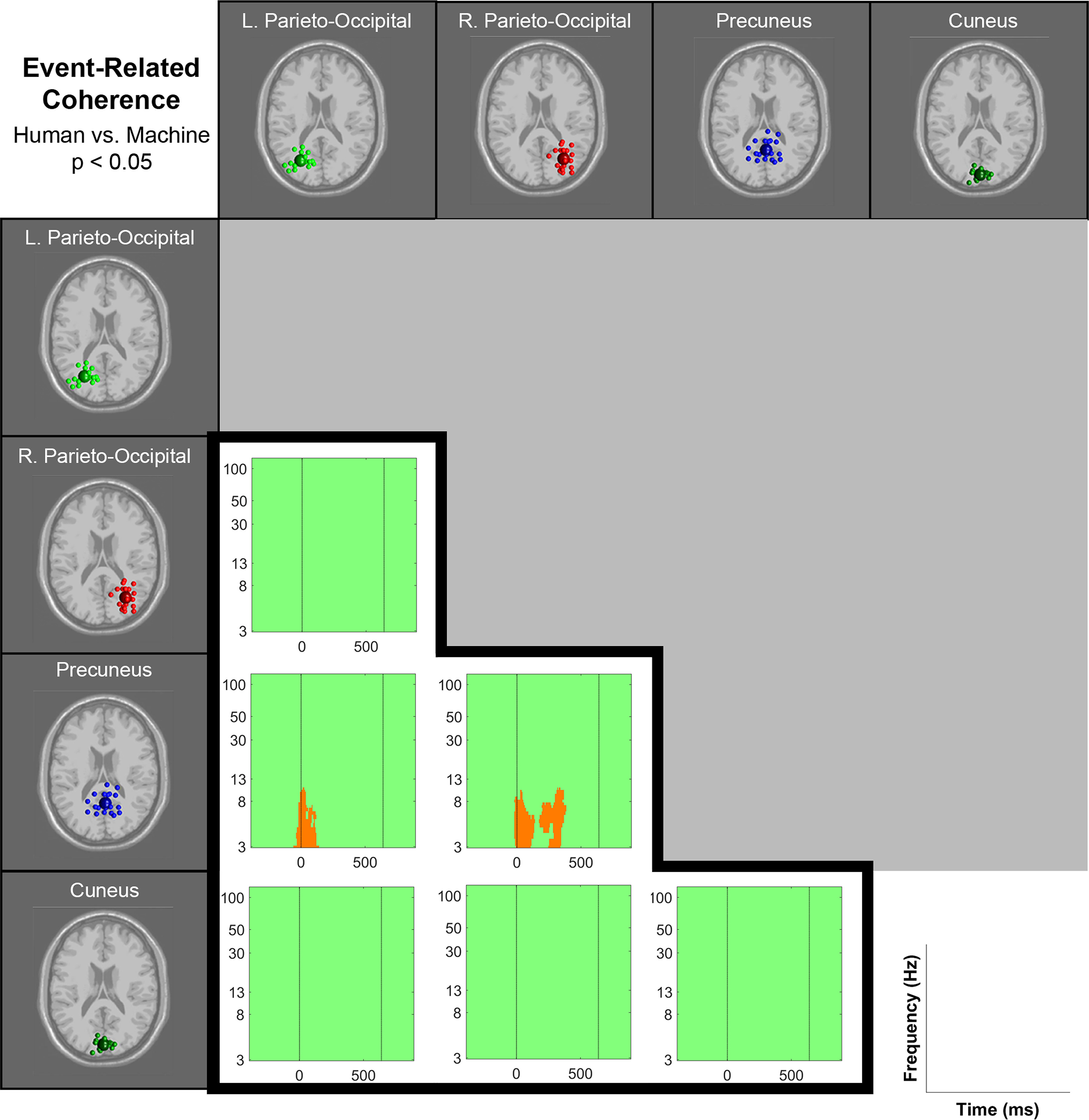
Significant differences in event-related coherence of parieto-occipital clusters between human player and ball machine trials (*p* < 0.05). At time 0, the ball appeared (either the human player made contact or the ball machine fed the ball). The data were not time-warped, but the average time of the participant hit across trials was overlaid (time 640 ms).

Event-related phase coherences (functional connectivity) between parieto-occipital clusters were found in θ, α, and γ frequency bands (Fig. 10). θ coherence appeared shortly after the ball’s appearance (time 0) and around 250 ms after the ball’s appearance. Although all of the pairwise coherence measures appeared stronger in the ball machine condition than the human player condition, only the coherence between precuneus and left/right parieto-occipital clusters showed statistically significant differences (Fig. 11). The coherence between precuneus and the left parieto-occipital cluster was stronger in the machine trials than the human trials in the θ frequency band right when the ball appeared to the participant (time 0). The coherence between precuneus and the right parieto-occipital cluster was stronger in the machine trials than the human trials in the θ frequency band right after the ball appeared to the participant (time 0) and around 250 ms after the ball’s appearance.

### Rally versus serve

**Figure 12.**
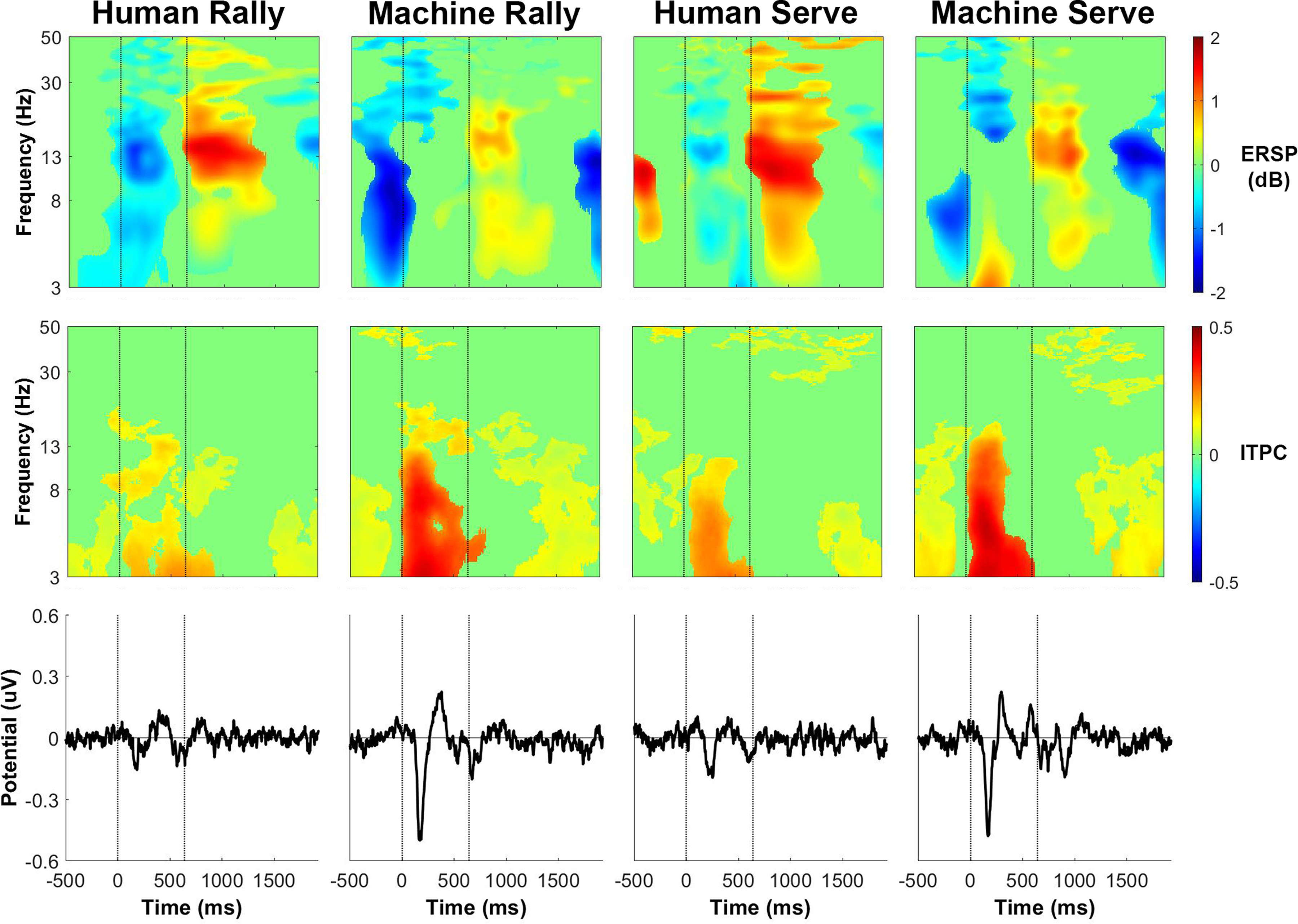
Left parieto-occipital cluster group average (*n* = 13) results of epochs of returning a rally ball (1 bounce) and returning a serve (2 bounces). Top row, Event-related spectral perturbation (ERSP) plots. Significant increases in spectral power relative to baseline are in red and significant decreases in spectral power relative to baseline are in blue (*p* < 0.05). Single trial spectrograms were warped to the event timing of the average swing cycle. At time 0, the ball appeared (either the human player made contact or the ball machine fed the ball). At 640 ms, the participant hit the ball. At 1924 ms the next ball appeared. Spectral baseline was the average power across the entire epoch. Middle row, Intertrial phase coherence (ITPC) plots, event-locked to when the ball appeared (time 0). Significant increases in intertrial phase coherence are in red (*p* < 0.05). Bottom row, Mean event-related potential (ERP) plots, event-locked to when the ball appeared (time 0). Intertrial phase coherence and event-related potentials were not warped to the hit events, but the same average event timings are overlaid.

**Figure 13.**
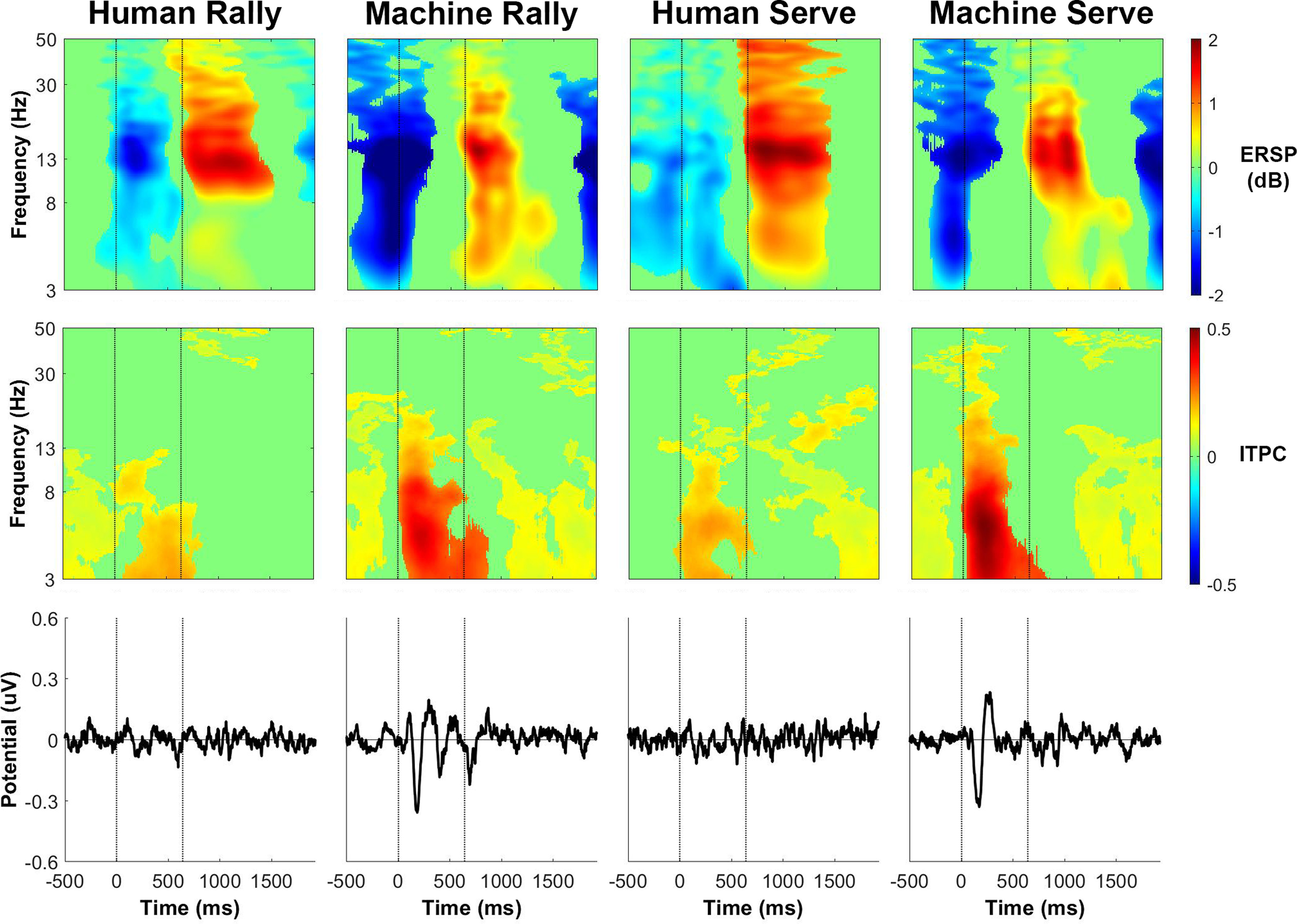
Right parieto-occipital cluster group average (*n* = 15) results of epochs of returning a rally ball (1 bounce) and returning a serve (2 bounces). Top row, Event-related spectral perturbation (ERSP) plots. Significant increases in spectral power relative to baseline are in red and significant decreases in spectral power relative to baseline are in blue (*p* < 0.05). Single trial spectrograms were warped to the event timing of the average swing cycle. At time 0, the ball appeared (either the human player made contact or the ball machine fed the ball). At 640 ms, the participant hit the ball. At 1924 ms, the next ball appeared. Spectral baseline was the average power across the entire epoch. Middle row, Intertrial phase coherence (ITPC) plots, event-locked to when the ball appeared (time 0). Significant increases in intertrial phase coherence are in red (*p* < 0.05). Bottom row, Mean event-related potential (ERP) plots, event-locked to when the ball appeared (time 0). Intertrial phase coherence and event-related potentials were not warped to the hit events, but the same average event timings are overlaid.

**Figure 14.**
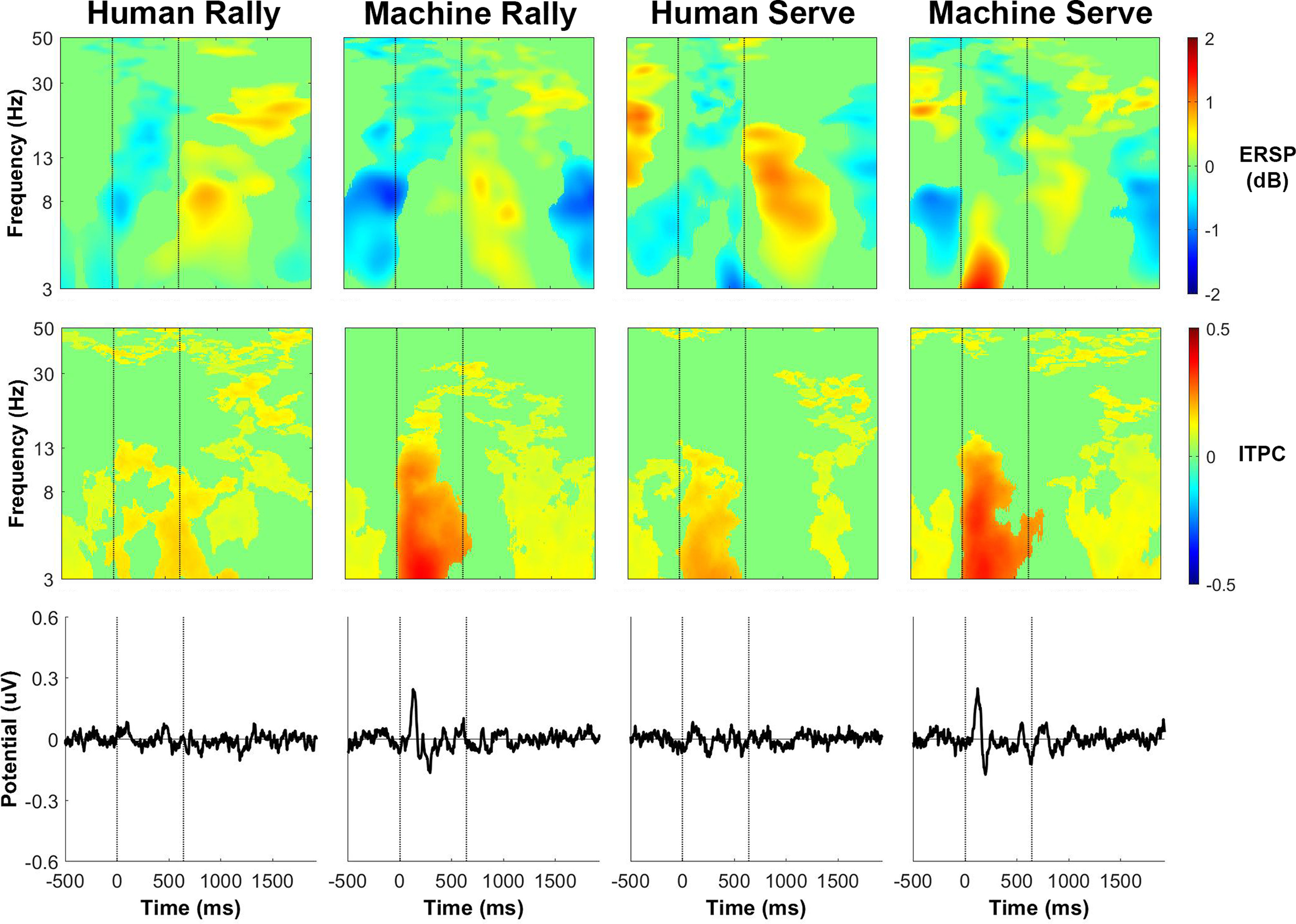
Precuneus (superior parietal) cluster group average (*n* = 17) results of epochs of returning a rally ball (1 bounce) and returning a serve (2 bounces). Top row, Event-related spectral perturbation (ERSP) plots. Significant increases in spectral power relative to baseline are in red and significant decreases in spectral power relative to baseline are in blue (*p* < 0.05). Single trial spectrograms were warped to the event timing of the average swing cycle. At time 0, the ball appeared (either the human player made contact or the ball machine fed the ball). At 640 ms, the participant hit the ball. At 1924 ms, the next ball appeared. Spectral baseline was the average power across the entire epoch. Middle row, Intertrial phase coherence (ITPC) plots, event-locked to when the ball appeared (time 0). Significant increases in intertrial phase coherence are in red (*p* < 0.05). Bottom row, Mean event-related potential (ERP) plots, event-locked to when the ball appeared (time 0). Intertrial phase coherence and event-related potentials were not warped to the hit events, but the same average event timings are overlaid.

**Figure 15.**
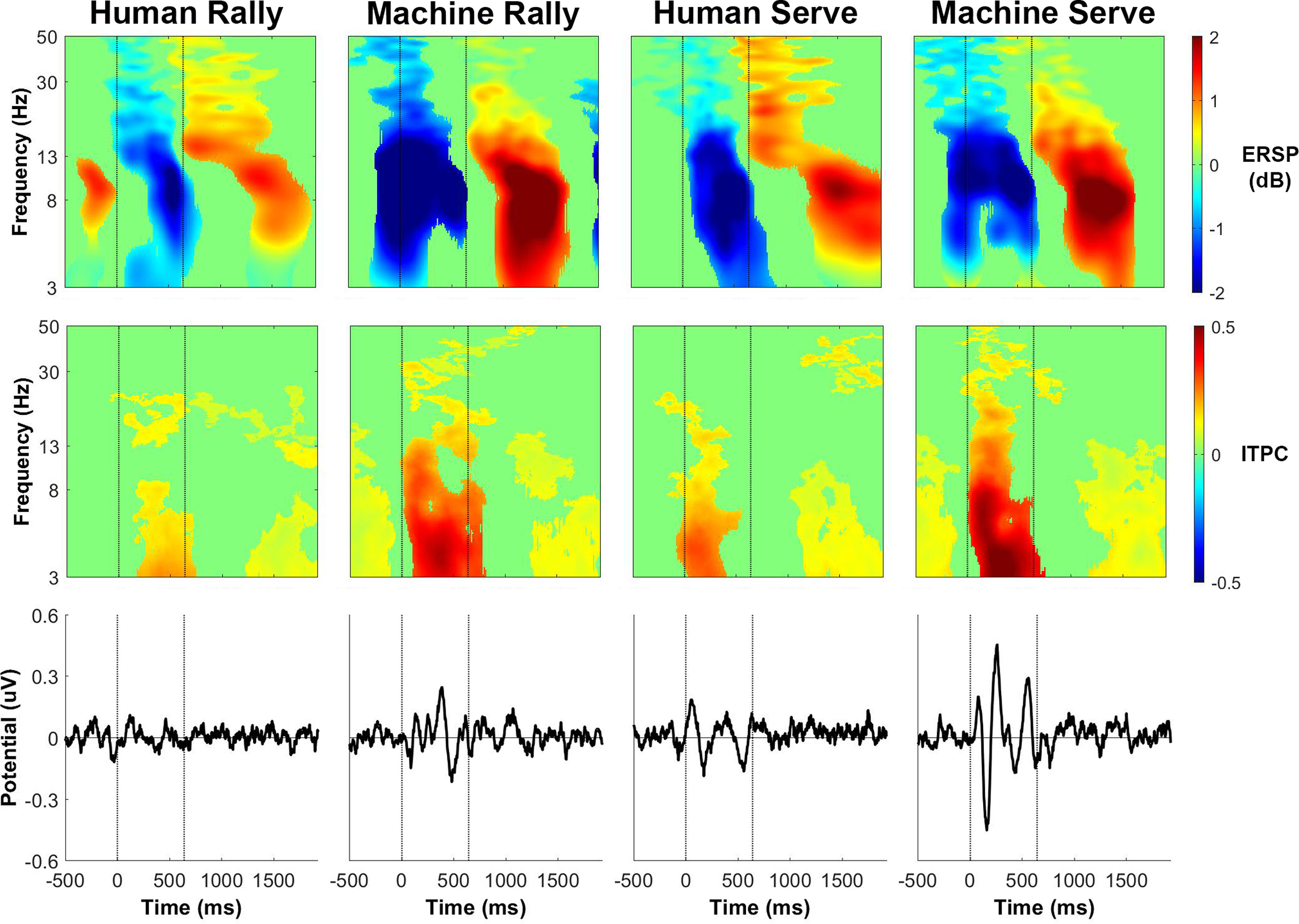
Cuneus (medial occipital) cluster group average (*n* = 13) results of epochs of returning a rally ball (1 bounce) and returning a serve (2 bounces). Top row, Event-related spectral perturbation (ERSP) plots. Significant increases in spectral power relative to baseline are in red and significant decreases in spectral power relative to baseline are in blue (*p* < 0.05). Single trial spectrograms were warped to the event timing of the average swing cycle. At time 0, the ball appeared (either the human player made contact or the ball machine fed the ball). At 640 ms, the participant hit the ball. At 1924 ms, the next ball appeared. Spectral baseline was the average power across the entire epoch. Middle row, Intertrial phase coherence (ITPC) plots, event-locked to when the ball appeared (time 0). Significant increases in intertrial phase coherence are in red (*p* < 0.05). Bottom row, Mean event-related potential (ERP) plots, event-locked to when the ball appeared (time 0). Intertrial phase coherence and event-related potentials were not warped to the hit events, but the same average event timings are overlaid.

The phase-locked activity, as shown by the intertrial phase coherence and event-related potential, were similar for receiving a rally ball or serve either from the ball machine or from the human player. Figures 12-15 show event-related activity of the clusters after separating epochs of the participant receiving a rally ball (one bounce) or receiving a serve (two bounces) from the human player and ball machine. The intertrial phase coherence for all clusters increased for θ and α bands for ∼500 ms after the ball feed in the ball machine trials that replicated both a rally ball and serve. There was less of an increase in intertrial phase coherence for either serves or rally balls in the human trials. The event-related potential also appeared at a similar timing of the increase in intertrial phase coherence for both ball machine conditions.

The event-related spectral perturbation plots for receiving a rally ball versus receiving a serve revealed differences. Previously, we showed that the left parieto-occipital and precuneus clusters had an increase in θ power between the ball machine feed and participant hit for ball machine trials only (Figs. 6 and 8). After separating epochs related to returning a rally ball on one bounce versus returning a serve on two bounces, the increase in θ only appeared while returning a serve from the ball machine (Figs. 12 and 14).

The broadband event-related desynchronization shifted earlier in the human serve trials than the in the human rally trials for the right parieto-occipital, precuneus, and cuneus clusters (Figs. 13-15). In the right parieto-occipital cluster (Fig. 13), θ, α, and β desynchronization occurred well before the human player serve, similar in timing to the ball machine trials. The shift in the human trials for the precuneus cluster (Fig. 14) was primarily in the θ frequency band. In the cuneus cluster (Fig. 15), the θ and α desynchronization still occurred after the human player serve, which was later than the ball machine trials. However, when compared with the human rally, the human serve condition’s α desynchronization shifted earlier.

## Discussion

We identified event-related dynamics in the parietal and occipital cortices that were tied to ball hit events during table tennis, a goal-oriented whole body motor task. In line with our main hypothesis, parieto-occipital clusters showed significant spectral power fluctuations in θ, α, and β bands that were consistent across an average swing cycle. We found differences in parieto-occipital electrocortical dynamics between human player and ball machine trials. Ball machine trials exhibited more fluctuations in θ power around the hit events than the human player trials. Additionally, all parieto-occipital clusters showed more phase coherence, an observable event-related potential, and an increase in event-related phase coherence during ball machine trials than human player trials.

There was significantly greater α power in the left parieto-occipital cortex for human player trials than ball machine trials (Fig. 5). Higher α power in the parieto-occipital cortex has been classically associated with cortical idling, a decrease in attention, or an attentional suppression mechanism (Neuper and Pfurtscheller, 2001; Iemi et al., 2022). Although somewhat counterintuitive, it is possible that ball machine trials demanded more attention and cognitive resources than human trials. The ball machine did not provide body language cues that participants could observe for ramping up their attention and motor response. As a result, returning a ball launched from a machine may require more focused attention than observing a human that gives natural motion cues about when the ball will be coming. Alternatively, the difference might have been in the novelty of hitting against the ball machine. It would be interesting to record data over many weeks of practice, both with a ball machine and a human opponent, to determine how electrocortical power fluctuations change with time.

In the event-related spectral perturbations (Figs. 6-9) and event-related coherences (Figs. 10, 11), the pattern of α power fluctuations and θ event-related coherence may be explained by the visuomotor demands of the parieto-occipital cortex during movement planning and execution. Previous studies show that a decrease in α power is common during motor planning and preparation (Van Der Werf et al., 2010b; Perfetti et al., 2011; Fumuro et al., 2015). After completing the movement, or in this case hitting the ball, the increase in α and β power (or “rebound”) likely reflects the sensorimotor network returning to baseline after executing a movement (Pfurtscheller et al., 1996). The increase in θ coherence between precuneus and left/right parieto-occipital clusters for the machine versus human conditions further supports the theory that ball machine trials demanded more cognitive resources than human trials. Evidence shows that an increase in interregional phase coherence is associated with controlling visuospatial attention (Lobier et al., 2018; Suess et al., 2021).

For all parieto-occipital clusters, ball machine trials had an observable event-related potential and higher intertrial phase coherence than human trials (Figs. 6-9). Hülsdünker and colleagues found a similar N2 motion-onset visual-evoked potential over electrodes near the MT+/V5 brain area (PO7, P7, P5, PO8, P8, and P6; Hülsdünker et al., 2020). Interestingly, they found a stronger N2 potential during a computer-based task used to elicit motion-onset visual-evoked potentials than during table tennis hits with a ball machine. It is possible that our parieto-occipital clusters’ event-related potentials could contribute to the N2 potential over these same electrodes. Another explanation of the phase-locked activity we observed is that the ball machine trials elicited more of “surprise” mechanism in the brain than the human trials. The N200 is commonly associated with error monitoring over fronto-central electrodes (Botvinick et al., 2001; Folstein and Van Petten, 2008). However, it has also been known to be diffuse over centro-parietal areas as well (Pezzetta et al., 2018). The lack of anticipatory cues from the ball machine may explain more of the surprise phenomena than the continuous back-and-forth nature of the task of hitting with a human player.

It is possible, but not certain, that the neural processes responsible for the phase-locked event-related potential contributed to the increased θ power in the event-related spectral perturbation (Yeung et al., 2004). However, θ power fluctuations and event-related potentials can occur at similar times without having the same causation. For example, Cohen and Donner found that the nonphase locked θ power over mid-frontal cortex was a better predictor of their conflict condition than the time-domain phase-locked event-related potential component (M.X. Cohen and Donner, 2013). In our study, the left parieto-occipital cluster (Fig. 6) and precuneus cluster (Fig. 8) both showed θ synchronization with similar timing as the increase in intertrial phase coherence and deflection in the event-related potential. Future research could study different behavioral task variations to determine whether the same neural mechanism is responsible for both observations.

An alternative explanation for the α and β event-related desynchronization we observed may be related to predictive coding and/or the action-observation phenomenon (Denis et al., 2017; Kilner et al., 2007; Yarrow et al., 2009). Research has shown that an expert athlete’s mirror neuron system activates when they observe familiar maneuvers that are part of their own skill repertoire. For example, experienced tennis players had earlier and greater μ and β (15–25 Hz) desynchronization in the sensorimotor cortex than novice tennis players during a tennis-related action observation and anticipation task (Denis et al., 2017). It is thought that the high outcome certainty (i.e., low prediction error) in these situations contributes to the action-observation phenomenon (Kilner et al., 2007; Tzagarakis et al., 2010). In our study, we observed event-related desynchronization during the motor preparation of striking the ball in both ball machine and human player conditions. However, we found greater α and β desynchronization for the ball machine compared with the human player conditions. The ball machine fed the ball at a more constant rate than the human player did, so it is possible that the low prediction error of the timing of the feed contributed to the earlier and greater desynchronization.

One of the differences between human player and ball machine trials was the continuity of the task. A rally with a human player is a continuous task. As the ball left the participant’s paddle, they were continuously monitoring the ball’s flight to the interception with the human player’s paddle, and consequently moving to intercept the next ball. While hitting from the ball machine, each hit was separate from the next hit. The ball machine did not move to intercept the hit from the participant. To control for this difference, we separated and analyzed epochs for participants after receiving a serve from the human player versus hitting a rally ball from the human player (Figs. 12-15). Receiving the serve could be likened to receiving a ball from the ball machine since there was no prior hit that the human player intercepted to hit back to the participant.

After separating the data into rally and serve epochs, spectral power fluctuations in the event-related spectral perturbations had differences across both rally versus serve hits and machine versus human hits. The θ synchronization in the left parieto-occipital and precuneus clusters were primarily found in the ball machine trials that replicated a serve (Figs. 12, 14). We also observed an earlier onset of event-related desynchronization when receiving serves from the human player in the right parieto-occipital, precuneus, and cuneus cortices compared with receiving a rally ball from the human player (Figs. 13-15). The greater magnitude in event-related desynchronization before the ball attack in the machine trials compared with the human trials suggests the participants were anticipating and preparing their movements earlier. The earlier shift in timing of the desynchronization before receiving the serve from a human player also supports this interpretation of motor preparation. In the ball machine trials, the direction that the machine was going to launch the ball was evident from the barrel of the machine, and the speed at which the ball was going to be launched was fairly consistent. In the human trials, there was little prior indication of the oncoming direction of the ball and there was a large variation in ball speed. These differences likely made it easier to prepare and plan for ball machine serves and rallies compared with human serves and rallies. Future work could use more advanced ball machines that could hide the trajectory of the ball and provide a better randomization of the ball speed. Based on the results, those two factors might explain some of the differences in electrocortical dynamics between machine and human returns.

Our study had several limitations that we have not yet mentioned. We recruited a wide variety of skill levels and included data from all the subjects in the analysis. Often, a focus of sport skill research is the comparison between expert and novice players. We did not have a skill level threshold for participation, so our results may be confounded by the skill level variation. According to Figure 1 and Extended Data Figure 1-1, our study was biased toward novice players whose experience was limited to games with friends and family. Given the neurophysiological adaptations between elite table tennis athletes and nonathletes (Wolf et al., 2014; Hülsdünker et al., 2019; Hughes et al., 2021; Yin et al., 2021), we would expect differences in electrocortical dynamics based on skill level. Future studies could replicate our experimental approach but with stricter inclusion criteria. Second, we did not separate successful and unsuccessful hits. Human and machine trials may elicit different error monitoring processes in the brain. While our dataset has the potential for analyzing hits by performance, we did not include that analysis here. Third, there are always alternative ways to perform group-level analyses in EEG data. We used k-means to cluster source-localized components from multiple participants as it is common and has provided robust results in past studies, but there are many alternative methods (Bigdely-Shamlo et al., 2013). Our approach also averaged many similar trials together, but this loses information in trial averages. Recent evidence supports that neural oscillations come in transient bursts of activity rather than sustained oscillations (van Ede et al., 2018; Vidaurre et al., 2018; Karvat et al., 2020). The precise timing of each burst may relate to the parameters of each event. For complex tasks like playing table tennis, it may be better to come up with alternative data analysis methods that focus on the entirety of the time series data and machine learning so that nuanced differences do not get lost in the average. Finally, we focused our analysis on parieto-occipital clusters, which limits our scope to the visual and perceptual brain processes. Future work should further investigate the frontal and sensorimotor brain regions that showed up as relevant to our table tennis task.

Analyzing high-density scalp EEG from human participants playing table tennis allowed us to examine the precise timing of electrocortical changes in the parieto-occipital cortices during a whole body visuomotor task with high ecological validity. Quantifying brain activity while the participants hit balls both from rallies and serves, and from a ball machine and a human player, revealed key differences in how and when the parietal cortex contributes to the visuomotor task. Event-related spectral perturbations revealed earlier broadband desynchronization and more evidence of phase-locked activity in ball machine trials than in human trials. The differences likely reflect how humans interpret body and machine cues regarding the trajectory and speed of the oncoming ball. It suggests that sport training with a machine may lead to different brain dynamics than training with humans. Future work could examine different anticipatory mechanisms involved with scenarios that are more complex.

## Synthesis

Reviewing Editor: Frederike Beyer, Queen Mary University of London Decisions are customarily a result of the Reviewing Editor and the peer reviewers coming together and discussing their recommendations until a consensus is reached. When revisions are invited, a fact-based synthesis statement explaining their decision and outlining what is needed to prepare a revision will be listed below. The following reviewer(s) agreed to reveal their identity: daniel Büchel, Avinash Singh.

Dear Ms Studnicki,

thank you for submitting your work to eNeuro, and I apologize for the delay in completing the initial review process. I now have comments from two independent reviewers, please see their comments below. Both reviewers commend the novelty and high technical quality of your work. However, they also raise some important points that need to be addressed before your manuscript can be considered for publication. In particular, please address the recommendations of reviewer #2 for including a more thorough discussion of relevant literature, as well as clarifying some important details in the methods section. Both reviewers further raise some points regarding the data analysis and handling of events and epochs, which need clarification.

Once your manuscript has been revised accordingly, please submit the revised version alongside an explanation of how the reviewers’ comments have been addressed.

With best wishes,

Frederike Beyer

Reviewer #1 comments

The work presented in the submitted manuscript entitled “Parieto-Occipital Electrocortical Dynamics during Real-World Table Tennis” provides novel insights into the cortical conitrbutions during the dynamic control of movement. The study is well designed, the data is carefully processed and the observations are discussed systematically.

The visual abstract is informative and clearly reflects the main findings of the study.

In the manuscript entitled “Parieto-Occipital Electrocortical Dynamics during Real-World Table Tennis”, the authors report observations from a well-designed mobile EEG study and provide novel insights into cortical contributions to dynamic goal-directed behavior in the context of sports. The analysis of EEG data extracted from a table tennis scenario provides evidence for the contribution of various parieto-occipital sources to dynamic sports behavior. Further, the authors report interesting observations of differences in cortical mechanisms related to simulated machine- based and interactive human-based performance of the motor task. The insights are methodlogically sound and provide one-of-its-king insights into sports-related snesorimtor control processes.

I congratulate the authors for their work. In the comments below, a feedback on the specific sections of the manuscript are provided, including minor concerns with regards to the content of the manuscript:

Intro:

The intro is well-written and concise and elaborates the scientific case investigated. Some minor comments concern:

- “ In fact, many 53 studies compared experts and non-athletes in simple laboratory tests to find which 54 neurophysiological adaptations are necessary for elite performance (Hughes et al., 2021; 55 Hülsdünker et al., 2019; Wolf et al., 2014; Yin et al., 2021).” Please specify, which adaptations were observed, since these are likely be associate with the acute neurophysiological mechanisms during table tennis play

- “However, their study focused on event-related potentials, which are neural responses 61 that contain only phase-locked, evoked activity. Their study may be missing information from the 62 non-phase-locked, induced, response (Pfurtscheller, 1992; Tallon-Baudry et al., 1996). “ Please elaborate while this could be relevant in a dynamic task like table tennis

- Towards the end of the study, a relevance statement/ application scenario would help to raise the importance of the conducted study. For instance, Gu et al (2019) or Yamasaki (2022) may provide some inspiration on the potential relevance of the description of table tennis as a stimulus evoking cortical activity

Gu et al :https://www.frontiersin.org/articles/10.3389/fpsyg.2019.01707/full

Yamasaki : https://www.mdpi.com/2673-8392/2/3/107

Materials & Methods

The methodological section is well-organized and provides a detailed overview on the conducted measures on the EEG. Due to the novelty of the study and the mobile setting, I would like to raise few minor concerns:

- “We recruited 37 participants (ages 23.5 +/- 6.7 years, 13 females) with a wide range of skill level and experience. “ I would like to encourage the authors to discuss whether the skill level could have been a factor influencing the results. Further, has any behavioral data (mistakes/ hits) been recorded to quantify skill differences and e.g. differentiate between succesfull and not-succesfull shots (in other word: good and bad motor control?) ?

- “Inertial measurement units (Cometa WaveTrack Inertial System, 2000 Hz) on the handles of two wooden paddles” Could you provide the information how the exact event timing was extracted from the IMU data ? At least for the responding player, I expect the data to quite noisy throughout the trial. Further, the more noise in detecting hits by humans than detecting shots from the machine might have effected the ERSP data reported later due to an inaccuracy of detecting the shot onset.

- “We recorded videos (GoPro Hero 7, range of 30-240 fps) for all trials to inspect the accuracy of hit events. We manually marked hits in the video using Adobe Premiere Pro software and aligned the events to the rest of the data using a cross-correlation with the acceleration from the inertial measurement units on the paddles.” - Can you provide any information on the gross accuracy of the event detection based on the IMUs ? Was there a systematic or random delay between true hit and actual hit ?

- “To avoid artificially inflating sample sizes, we retained a single component per participant in each cluster that explained the greatest amount of scalp variance. On average, nine components were removed per cluster.” Please briefly explain how scalp variance was calculated.

- “We epoched the data 1.0 to 2.5 seconds around the time when the ball appeared to the participant and rejected 10% of epochs that had the highest voltage maxima, resulting in (mean +/- SD) 546 +/- 144 epochs with the ball machine and 543 +/- 111 epochs with the human player”. Again, I would be curious about information on the accuracy of appearance detections between ball and human conditions. I am aware of the limitations in recording events from real-world behavior, but would at least like to see this issue discussed. Further, I would like to know whether the differences in HIT frequency were considered for analysis ? As seen in the video attached, the hitting frequency was higher for the human than for the machine condition. Considering an epoch length of 3.5 seconds, is there the chance that two consecutive trials overlapped for one epoch analyzed (especially for the rally condition) ?

Results:

The results are concise and are further supported by well-chosen visualizations.

Discussion:

The discussion is well-structured and focusses on the most important findings. I only have few minor points to raise:

- “After separating the data into rally and serve epochs, spectral power fluctuations in the event related spectral perturbations had differences across both rally vs. serve hits and machine vs. human hits. The theta synchronization in the left parieto-occipital and precuneus clusters were primarily found in the ball machine trials that replicated a serve (Figures 10 and 12)” Please discuss how overlapping events may have affected the findings, particularly for the human rallye condition showing a less-clear ERSP and ERP.”

- Please add to the limitations part that the restriction towards parieto-occipital brain regions limited the analysis towards the perceptual aspects of the table tennis task. Insights into processes related to the frontal- and motor cortices may provide complementary insights into the task.

Reviewer #2 comments

This is a novel work in mobile EEG in a real-world task and definitely provides a greater inside of brain dynamics shown in the author’s results.

Visual abstract looks informative however highly technical and can be improved with reduced technicality with simplified drawings for ERSP and ITPC figures mentioned in the abstract.

In this work, the author presented an exploratory electrocortical dynamics for a parietal-occipital network in real-world table tennis using EEG and MR. This is a great piece of work and a well-written method section to replicate the study elsewhere. There are only minor concerns that can further improve the presented work. Please see the comments below:

Introduction

Although well-written introduction, however, motivation could be stronger to understand. I can see that authors are interested in exploring parietal-occipital cortices but do not quite justify why only this region of the brain but not others. Also, it seems that there are several research works are missing which are highly essential toward related to hand movement to perform a real-world or virtual kind of a task

Materials

#89 It would be good if the author report the reason behind choosing 37 participant?

#109 Can the author add more information on what is TTL pulses?

#113 It still needs to be clarified how event are aligned using cross-correlation. It would be great if the author add more detail to it ?

I enjoyed reading the remaining part of the method section with careful and all detail systematically.

There seem to few references are missing, such as #158 for iCanClean and it would be great to understand/add a reference for TTL Synchronisation process

#237 How many participants were chosen in total for functional connectivity?

#256 I am a bit unsure of how to downsample the trials, more details are needed?

Discussion

#383-394 I do not quite agree with the argument that the body language of machines may be causing the higher alpha power, although the author’s reason sounds valid. Does this argument supported by any previous research? I wonder if it is simply due to the action-observation phenomenon.

#412 This part of the explanation also seems to relate to the previous argument action-obersevation phenomenon, where machine’s motion does not quite match with normal motion involved with humans. It would be great to look at how well ACC responds in these trials with a machine and will provide additional support to finding and explanation behind N2.

## Author Response

REVIEWER #1

Reviewer #1, Comment #1: “In fact, many 53 studies compared experts and non-athletes in simple laboratory tests to find which 54 neurophysiological adaptations are necessary for elite performance (Hughes et al., 2021; 55 Hülsdünker et al., 2019; Wolf et al., 2014; Yin et al., 2021).” Please specify which adaptations were observed, since these are likely to be associated with the acute neurophysiological mechanisms during table tennis play.

Author Response:

The following neurophysiological adaptations were observed in elite table tennis players (compared to non-athletes):

-Better dynamic visual acuity, wider visual field, and better recognition of peripheral targets (although not a huge difference); and faster psychomotor responses (simple reaction time, choice response time, hand movement time) (Hughes et al., 2021)

-Better reaction time, earlier stimulus-locked N2 potential in middle temporal visual area (MT), earlier EMG onset relative to N2 potential – which probably reflects a more efficient integration of visual information (Hulsdunker et al., 2019)

-Stronger 8-10 Hz sensorimotor rhythm event-related desynchronization in elite athletes than in amateurs while watching film of a serve; weaker event-related desynchronization in the fronto-parietal cortex with higher world rank points among the experts (Wolf et al., 2014)

-Increased intrinsic functional flexibility in sensorimotor areas and increased intrinsic functional specialization in visual areas for experts compared to non-athletes (Yin et al., 2021)

Manuscript edits:

We included more details of elite table tennis athletes'{trade mark, serif} neurophysiological adaptations in the introduction (lines 46-48).

Reviewer #1, Comment #2: “However, their study focused on event-related potentials, which are neural responses 61 that contain only phase-locked, evoked activity. Their study may be missing information from the 62 non-phase-locked, induced, response (Pfurtscheller, 1992; Tallon-Baudry et al., 1996).” Please elaborate why this could be relevant in a dynamic task like table tennis.

Author Response:

In this section, we were trying to communicate the benefit of other data visualization techniques besides the event-related potential. ERP analyses have been the predominant way to analyze EEG data since the middle of the 20th century. Scientists compute ERPs by averaging response epochs that are time-locked to an event of interest. However, the brain activity that is not exactly synchronized in time AND phase to the experimental events cancels out in the average – only the time-locked and phase-locked data that remains comprises the event-related potential. However, studies show that useful brain dynamics lie within the non-phase locked brain rhythms (Van Dijk et al., 1992) (Makeig, 1993).

For our table tennis task, we do not think the induced, non-phase locked information is a better metric because the sport is dynamic. Rather, the picture may be incomplete if we only consider the phase locked ERP approach. Since the Hulsdunker 2020 study only used channel-based ERPs, we wanted to provide a different view that considers both phase-coherent and phase-incoherent data.

-Van Dijk et al., 1992: https://pubmed.ncbi.nlm.nih.gov/1376665/

-Makeig, 1993: https://sccn.ucsd.edu/∼scott/ersp93.html

Manuscript edits:

We included an explanation of the limitations of ERP analysis (lines 57-59).

Reviewer #1, Comment #3: Towards the end of the study, a relevance statement/ application scenario would help to raise the importance of the conducted study. For instance, Gu et al (2019) or Yamasaki (2022) may provide some inspiration on the potential relevance of the description of table tennis as a stimulus evoking cortical activity

Gu et al :https://www.frontiersin.org/articles/10.3389/fpsyg.2019.01707/full

Yamasaki : https://www.mdpi.com/2673-8392/2/3/107

Author Response:

Thank-you for these articles!

Manuscript edits:

We added this information to the paragraph in the introduction that discusses table tennis (lines 48-52).

Reviewer #1, Comment #4A: “We recruited 37 participants (ages 23.5 +/- 6.7 years, 13 females) with a wide range of skill level and experience.” I would like to encourage the authors to discuss whether the skill level could have been a factor influencing the results.

Author Response:

It is possible that the skill level of the participants influenced our results. There are well-documented differences in neurophysiological adaptations between elite table tennis athletes and non-athletes (faster visuomotor responses, reaction time, and efficient integration of visual information). However, we recruited a wide range of skill level and experience – so we agree that we should better report any biases in skill level. Overall, the spread of skill level was biased towards novice/less-experienced players, although all participants could consistently make contact with the ball. Most participants were the “typical garage-level player”.

Manuscript edits:

We included the breakdown of responses to the skill level questionnaire in a Figure and in an Extended Data Figure so readers have a better idea of our participants'{trade mark, serif} skill level / experience distribution. We also added to the Discussion how skill level may have affected our results (lines ∼488).

Reviewer #1, Comment #4B: Further, has any behavioral data (mistakes/ hits) been recorded to quantify skill differences and e.g., differentiate between successful and not-successful shots (in other word: good and bad motor control?)?

Author Response:

Yes! We manually marked the performance of each hit by reviewing the GoPro video data for all trials and all participants. In this analysis, we included all successful and unsuccessful hits in our averages. In the future, we may separate hits by performance – an excellent idea for a study! Here, we thought a performance analysis would be beyond the scope of the article.

Manuscript edits:

We added a point in the discussion/limitation section (lines ∼497) that errors/successful hits would be a valuable study in the future.

Reviewer #1, Comment #5: “Inertial measurement units (Cometa WaveTrack Inertial System, 2000 Hz) on the handles of two wooden paddles” Could you provide the information how the exact event timing was extracted from the IMU data? At least for the responding player, I expect the data to be quite noisy throughout the trial. Further, the more noise in detecting hits by humans than detecting shots from the machine might have affected the ERSP data reported later due to an inaccuracy of detecting the shot onset.

Author Response:

The hit events were marked when the first derivative of the resultant acceleration from the IMUs exceeded 0.75 gravity. Yes, you are correct that the IMUs on the paddles were “noisier” than the IMU on the ball machine. However, the impact of the ball with the paddle still provided easily detectable peaks (see Figure 3 + Extended Data Figure 3-1 in the revised manuscript). There may have been some errors in the event marking (like in the ball machine IMU ∼7 seconds in Figure 3, lower left). However, these small errors should average out.

Manuscript edits:

We updated the language in the methods section and included an additional Figure + Extended Data Figure, which shows this explanation.

Reviewer #1, Comment #6: “We recorded videos (GoPro Hero 7, range of 30-240 fps) for all trials to inspect the accuracy of hit events. We manually marked hits in the video using Adobe Premiere Pro software and aligned the events to the rest of the data using a cross-correlation with the acceleration from the inertial measurement units on the paddles.” - Can you provide any information on the gross accuracy of the event detection based on the IMUs? Was there a systematic or random delay between true hit and actual hit?

Author Response:

The GoPro video data did not give us timing of the “true hits” (too low of a sampling rate). Rather, the GoPro video allowed us to look back and determine how many events we should find and general timing of the hit events. The IMU data (sampling frequency = 2000 Hz) had a sufficient sampling rate to provide high precision of the hit events (gets rid of any random delays).

There could have been systematic delays if the IMU Cometas sensors were not well synchronized to the EEG BrainVision data. However, we checked the sensor synchronization by placing a Cometas IMU on top of the BrainVision LiveAmp amplifier (which has a built-in accelerometer sensor) for a subset of the participants. By comparing the acceleration from both systems, we were able to determine that the data was synchronized properly.

Manuscript edits:

N/A

Reviewer #1, Comment #7: “To avoid artificially inflating sample sizes, we retained a single component per participant in each cluster that explained the greatest amount of scalp variance. On average, nine components were removed per cluster.” Please briefly explain how scalp variance was calculated.

Author Response:

The scalp variance came from the dipole fitting approach while solving the EEG inverse problem. Here, the scalp variance described how well the dipole model explained the measured scalp topography. Another way to think about it – a high scalp variance means that the component had a very “dipolar” decomposition (Delorme et al., 2012). The difference between the single dipole model and the true measured scalp topography is known as the “residual variance” (1 – scalp variance). The computation of the scalp variance was carried out in the EEGLab function DIPFIT 3.3.

-Delorme et al., 2012: https://www.ncbi.nlm.nih.gov/pmc/articles/PMC3280242/

-Other useful references:

https://www.fieldtriptoolbox.org/workshop/baci2017/inverseproblem/

https://eeglab.org/tutorials/09_source/DIPFIT.html

Manuscript edits:

We included a brief explanation in line 203.

Reviewer #1, Comment #8: “We epoched the data 1.0 to 2.5 seconds around the time when the ball appeared to the participant and rejected 10% of epochs that had the highest voltage maxima, resulting in (mean +/- SD) 546 +/- 144 epochs with the ball machine and 543 +/- 111 epochs with the human player”. Again, I would be curious about information on the accuracy of appearance detections between ball and human conditions. I am aware of the limitations in recording events from real-world behavior but would at least like to see this issue discussed. Further, I would like to know whether the differences in HIT frequency were considered for analysis. As seen in the video attached, the hitting frequency was higher for the human than for the machine condition. Considering an epoch length of 3.5 seconds, is there the chance that two consecutive trials overlapped for one epoch analyzed (especially for the rally condition)?

Author Response:

To your first point, there was not a big difference in event marking accuracy between ball machine and human conditions (refer to Figure*). In fact, the video data allowed us to reject any events that were outside the window of manually marked hit events from the Adobe Premiere software. In lab-based studies, it would probably be sufficient to use only one method for marking events. However, given the “messiness” of recording events in the real world, we used multiple methods for marking events.

To your second point, it is true that the hitting frequency was higher for human (every 1.4 seconds) than ball machine trials (every 2 seconds). We chose an epoch length of 3.5 seconds to capture all the events for the ball machine conditions. In the quick (human) epochs, we did have overlapping events, but this did not affect our outcome measures. In an earlier analysis, we compared ERSP for just ball machine, just human, and then combined together. There were no appreciable differences between the ERSPs across the 3 analyses. This indicates that the frequency of the hit events did not affect the spectral baseline subtraction.

Manuscript edits:

N/A

Reviewer #1, Comment #9: “After separating the data into rally and serve epochs, spectral power fluctuations in the event related spectral perturbations had differences across both rally vs. serve hits and machine vs. human hits. The theta synchronization in the left parieto-occipital and precuneus clusters were primarily found in the ball machine trials that replicated a serve (Figures 10 and 12)” Please discuss how overlapping events may have affected the findings, particularly for the human rally condition showing a less-clear ERSP and ERP.”

Author Response:

We do not believe that overlapping events affected our findings (see reasoning from Comment #8).

Manuscript edits:

N/A

Reviewer #1, Comment #10: “Please add to the limitations part that the restriction towards parieto-occipital brain regions limited the analysis towards the perceptual aspects of the table tennis task. Insights into processes related to the frontal- and motor cortices may provide complementary insights into the task.

Author Response:

Good point. We did not want to expand our analysis to all the brain regions for brevity'{trade mark, serif}s sake. However, our conclusions were limited to visual and perceptual brain processes –; as you point out.

Manuscript edits:

We updated the limitation section in the manuscript : line ∼509.

REVIEWER #2

Reviewer #2, Comment #1: Although a well-written introduction, however, motivation could be stronger to understand. I can see that authors are interested in exploring parietal-occipital cortices but do not quite justify why only this region of the brain but not others. Also, it seems that several research works are missing which are highly essential, related to hand movement to perform a real-world or virtual kind of task.

Author Response:

Thank you for the feedback, we attempted to clarify our motivation.

Manuscript edits:

We added to our introduction (lines 72-74), and we included additional references related to hand movement studies (first paragraph in introduction).

Reviewer #2, Comment #2: #89 It would be good if the author reported the reason behind choosing 37 participants?

Author Response:

We aimed to have ∼25 participants left in the analysis (after removing some due to technical limitations – not good enough contact between the electrode and scalp, too much artifact, etc.). We collected 37 participants to have a “buffer”, knowing we would have to discard some of the data if it was too noisy.

Manuscript edits:

We included a reference for our rationale under Methods > Participants (Szucs and Ionnidis, 2020).

Reviewer #2, Comment #3: #109 Can the author add more information on what is TTL pulses?

Author Response:

TTL stands for transistor-transistor logic; bipolar transistor circuits that switch on/off to maintain logic states, sending digital pulses to equipment for offline synchronization. TTL pulses are one way to accomplish synchronization between multiple devices.

In the case of our experiment, we used an Arduino Timer Module that sent our sync signal to both EEG and IMU systems at the same time. In post-processing, we aligned the sync signals from both systems to synchronize them. However, we are glad you brought attention to this. We actually sent TTL pulses (digital) to the BrainVision EEG LiveAmps, but we sent analog input to the Cometas IMUs (a slight technical clarification). The Cometas system contains both IMU and EMG sensors. Similar to the approach from Artoni et al, 2018 (Figure 1D), we sent TTL to the EEG BrainVision amplifiers and an analog input to an EMG Cometas electrode at the same time.

Artoni et al., 2018: https://www.frontiersin.org/articles/10.3389/fnhum.2017.00652/full

Manuscript edits:

Updated the Methods section, lines 111-113.

Reviewer #2, Comment #4: #113 It still needs to be clarified how events are aligned using cross-correlation. It would be great if the author added more detail to it.

Author Response:

Relative hit timings were manually marked in the videos using Adobe Premiere Pro software and exported as excel documents (for each individual trial), giving us a list of event timings relative to the first hit event (which we assigned as “time 0”). To better automate the alignment of the first hit event to the first hit from the paddle IMU data, we used the xcorr function in Matlab to cross correlate the two signals. The reason the cross-correlation worked was that each stint of hits were different lengths. Even in the ball machine trials, there were typically a few shots where the ball machine was “jammed” (missed feeding a ball). These breaks in the hits made it easy to line up the two time series. This process was interactive, and we double-checked that the alignment was accurate.

Manuscript edits:

Updated the Methods section, lines 115-118.

Reviewer #2, Comment #5: There seem to few references are missing, such as #158 for iCanClean and it would be great to understand/add a reference for TTL Synchronization process

Author Response:

We intentionally omitted the reference for iCanClean to remain anonymous. We will send the reference to the editor if/when the article finishes the review process.

Manuscript edits:

Reference for the synchronization process has been updated (Artoni et al., 2018) â€” line 113.

Reviewer #2, Comment #6: #237 How many participants were chosen in total for functional connectivity?

Author Response:

Mean +/- SD = 14 +/- 2 participants

Manuscript edits:

Updated Methods section to include mean +/- SD (line 244).

We also updated the Figure 8 caption to include how many participants were chosen for each cluster pairwise functional connectivity.

Reviewer #2, Comment #7: #256 I am a bit unsure of how to down sample the trials, more details are needed?

Author Response:

We meant that we randomly sampled the epochs per participant per condition so that the number of epochs were similar across conditions. Human serves had the least number of epochs (67 +/- 16 human serves). We wanted all the other conditions (hit types; like machine rallies, machine serves, human rallies) to have a similar number of epochs to compare.

Manuscript edits:

Updated language in methods section, lines 263-265.

Reviewer #2, Comment #8: #383-394 I do not quite agree with the argument that the body language of machines may be causing higher alpha power, although the author’s reason sounds valid. Is this argument supported by any previous research? I wonder if it is simply due to the action-observation phenomenon.

Author Response:

It is difficult to determine precisely what caused the higher alpha/beta power in the human player vs. ball machine trials. We discussed several of the potential theories... however; you bring up an additional good theory – the action-observation phenomenon. Here are our thoughts:

An alternative explanation for the alpha and beta event-related desynchronization we observed may be related to predictive coding and/or the action-observation phenomenon (Denis et al., 2017; Kilner et al., 2007; Yarrow et al., 2009). Research has shown that an expert athlete'{trade mark, serif}s mirror neuron system activates when they observe familiar maneuvers that are part of their own skill repertoire. For example, experienced tennis players had earlier and greater mu and beta (15-25 Hz) desynchronization in the sensorimotor cortex than novice tennis players during a tennis-related action observation and anticipation task (Denis et al., 2017). It is thought that the high outcome certainty (i.e. low prediction error) in these situations contributes to the action-observation phenomenon (Kilner et al., 2007; Tzagarakis et al., 2010). In our study, we observed event-related desynchronization during the motor preparation of striking the ball in both ball machine and human player conditions. However, we found greater alpha and beta desynchronization for the ball machine compared to the human player conditions. The ball machine fed the ball at a more constant rate than the human player did, so it is possible that the low prediction error of the timing of the feed contributed to the earlier and greater desynchronization.

Denis, 2017: https://www.sciencedirect.com/science/article/pii/S1053811916305766

Kilner, 2007: https://link.springer.com/article/10.1007/s10339-007-0170-2

Yarrow, 2009: https://www.nature.com/articles/nrn2672

Tzagarakis, 2010: https://www.frontiersin.org/articles/10.3389/fnins.2015.00246/full#B54

Manuscript edits:

We included this in our discussion (lines 446-459).

Reviewer #2, Comment #9: #412 This part of the explanation also seems to relate to the previous argument action-observation phenomenon, where machine’s motion does not quite match with normal motion involved with humans. It would be great to look at how well ACC responds in these trials with a machine and will provide additional support to finding an explanation behind N2.

Author Response:

We really wanted to focus and hone in on the parieto-occipital cortices for this analysis. We agree that the anterior cingulate / frontal clusters may yield useful insight into why the ERP waveform was much larger for the ball machine trials than the human trials, so we included figures in Extended Data Figure 9-1 of the ERSP/ITPC/ERP plots of all the other source-localized clusters.

Manuscript edits:

Extended Data Figure 9-1 and report in the results, lines 374-379.
