# Supplementary figures and images for: Parieto-Occipital Electrocortical Dynamics during Real-World Table Tennis
Source: eNeuro. 2023 Apr 13;10(4):ENEURO.0463-22.2023. doi: 10.1523/ENEURO.0463-22.2023 (PMC10158585; doi:10.1523/ENEURO.0463-22.2023)

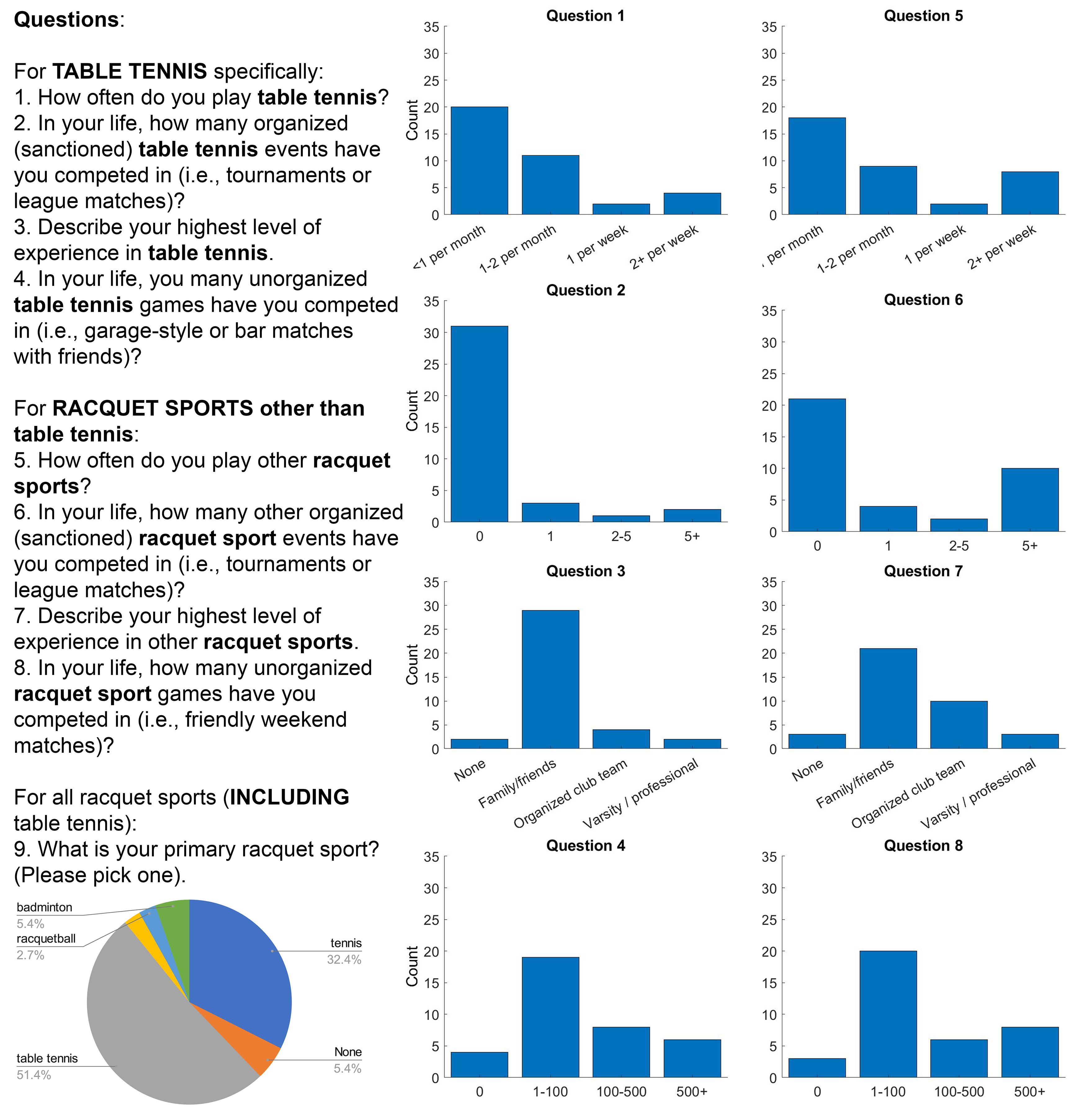

Supplement: Extended Data Figure 1-1 — Results from the full survey for gauging participant’s skill level and experience in table tennis and racquet sports. Download Figure 1-1, TIF file. [file enu-eN-NWR-0463-22-s03.tif]

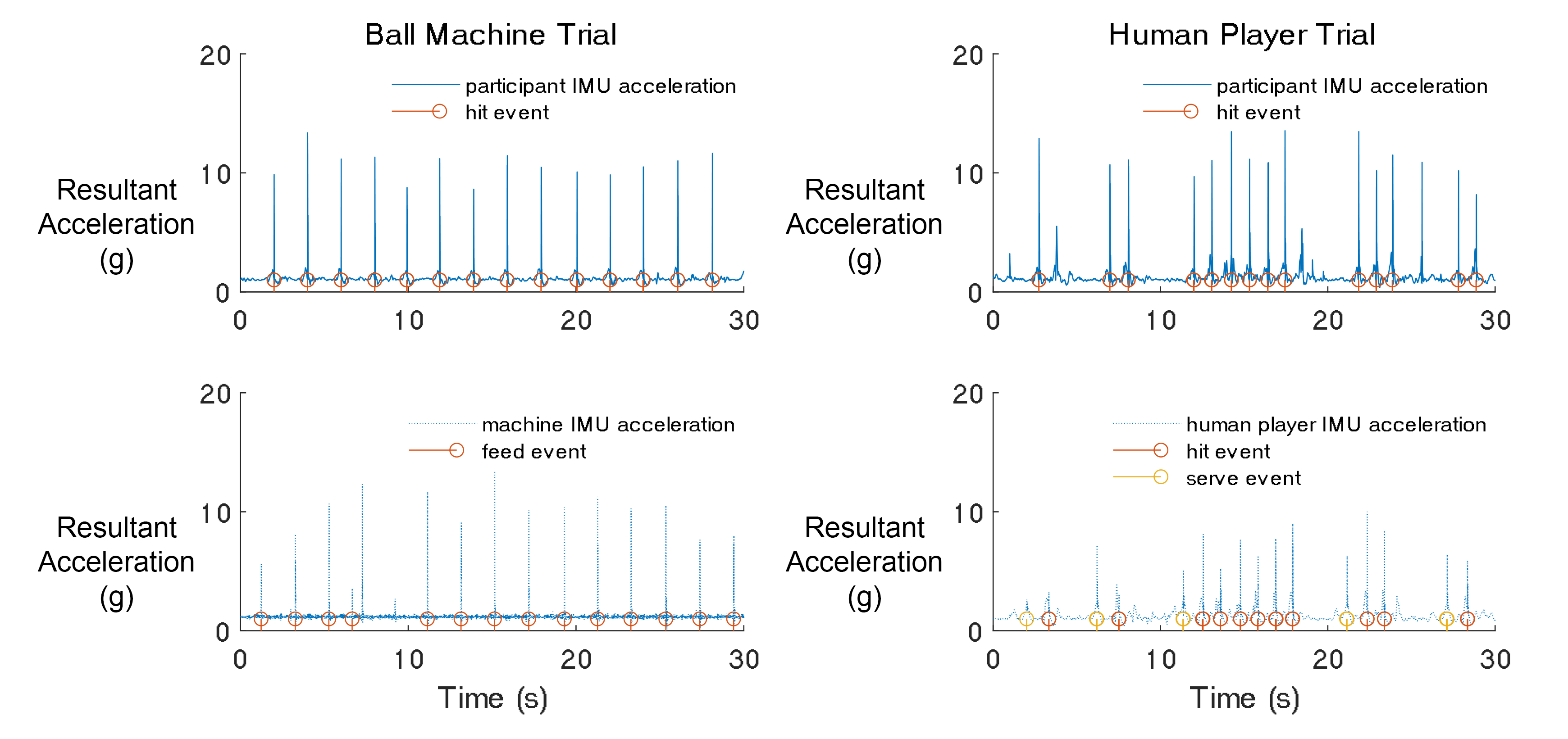

Supplement: Extended Data Figure 3-1 — Exemplary IMU data from a single participant. The blue traces show the resultant acceleration of the IMUs. The orange stem markers show the marked hit events (either ball machine feeds, human player hits, or participant hits). The yellow markers show the serve hit events. The acceleration data was sufficiently clean to extract the timing of hit events. There were some errors in event marking (like in the ball machine IMU data ∼7 s), but these errors were uncommon. Download Figure 3-1, TIF file. [file enu-eN-NWR-0463-22-s05.tif]

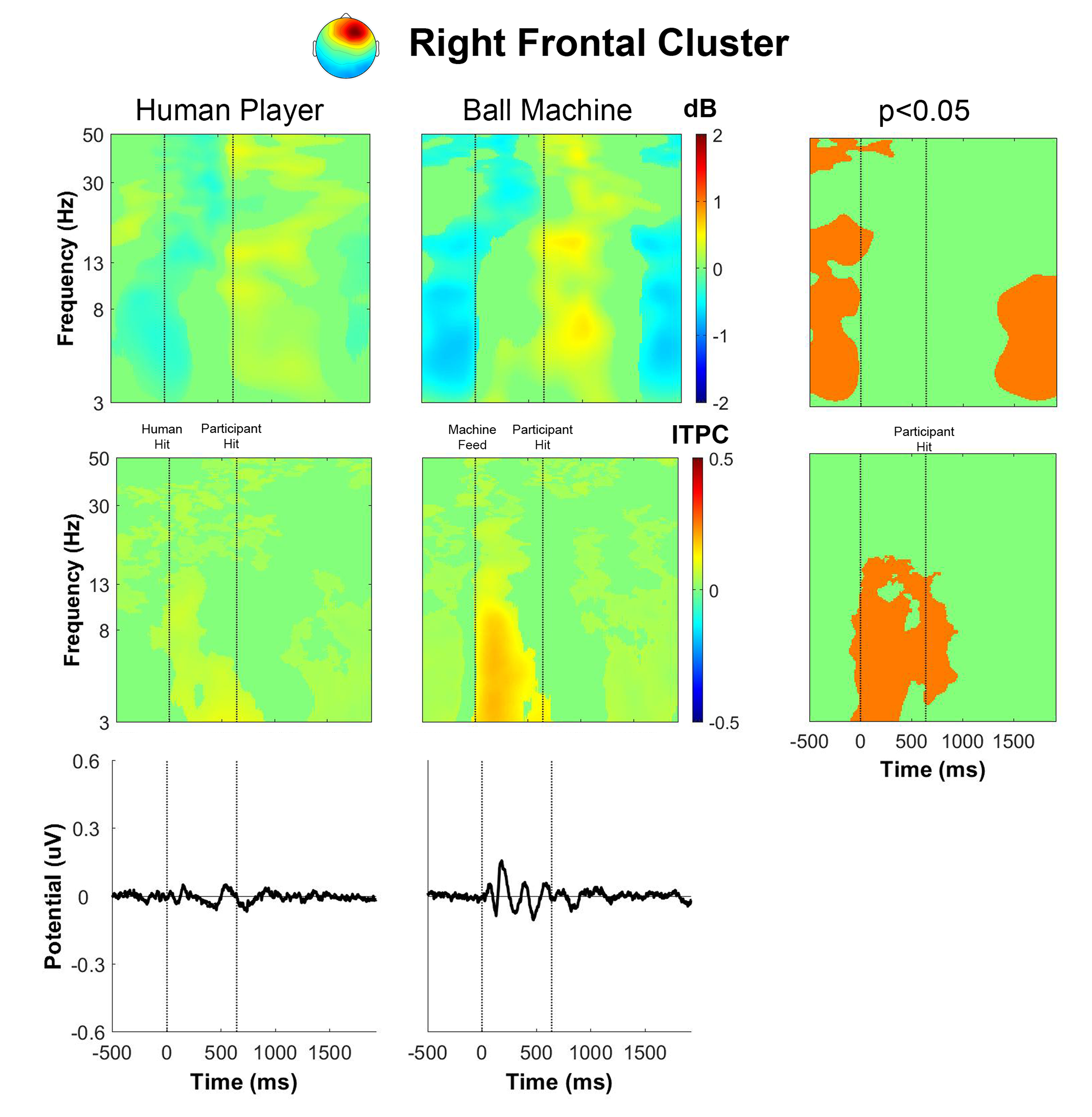

Supplement: Extended Data Figure 9-1 — Right frontal cluster group average (n = 18) results. Download Figure 9-1, TIF file. [file enu-eN-NWR-0463-22-s06.tif]

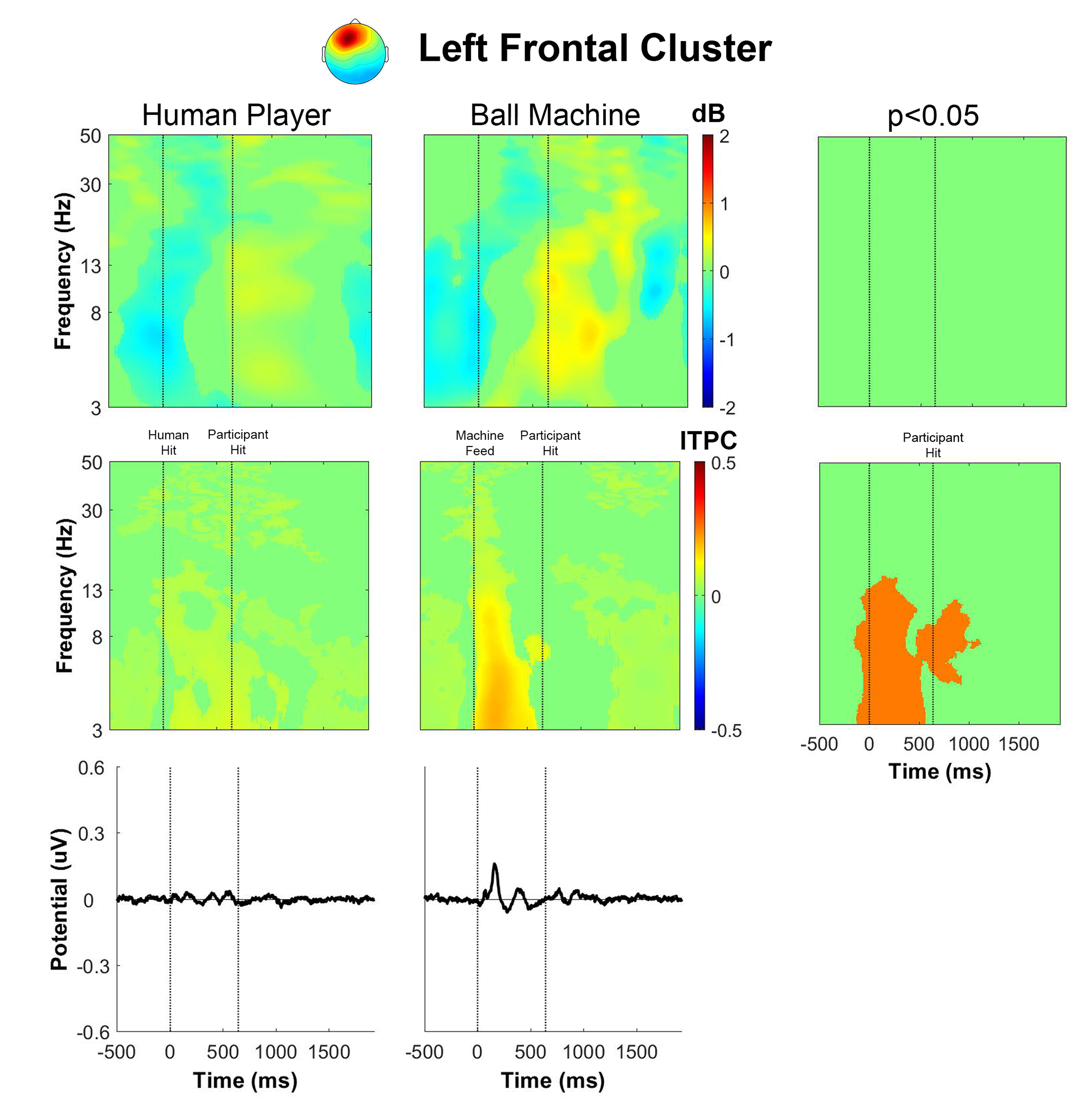

Supplement: Extended Data Figure 9-2 — Left frontal cluster group average (n = 18) results. Download Figure 9-2, TIF file. [file enu-eN-NWR-0463-22-s07.tif]

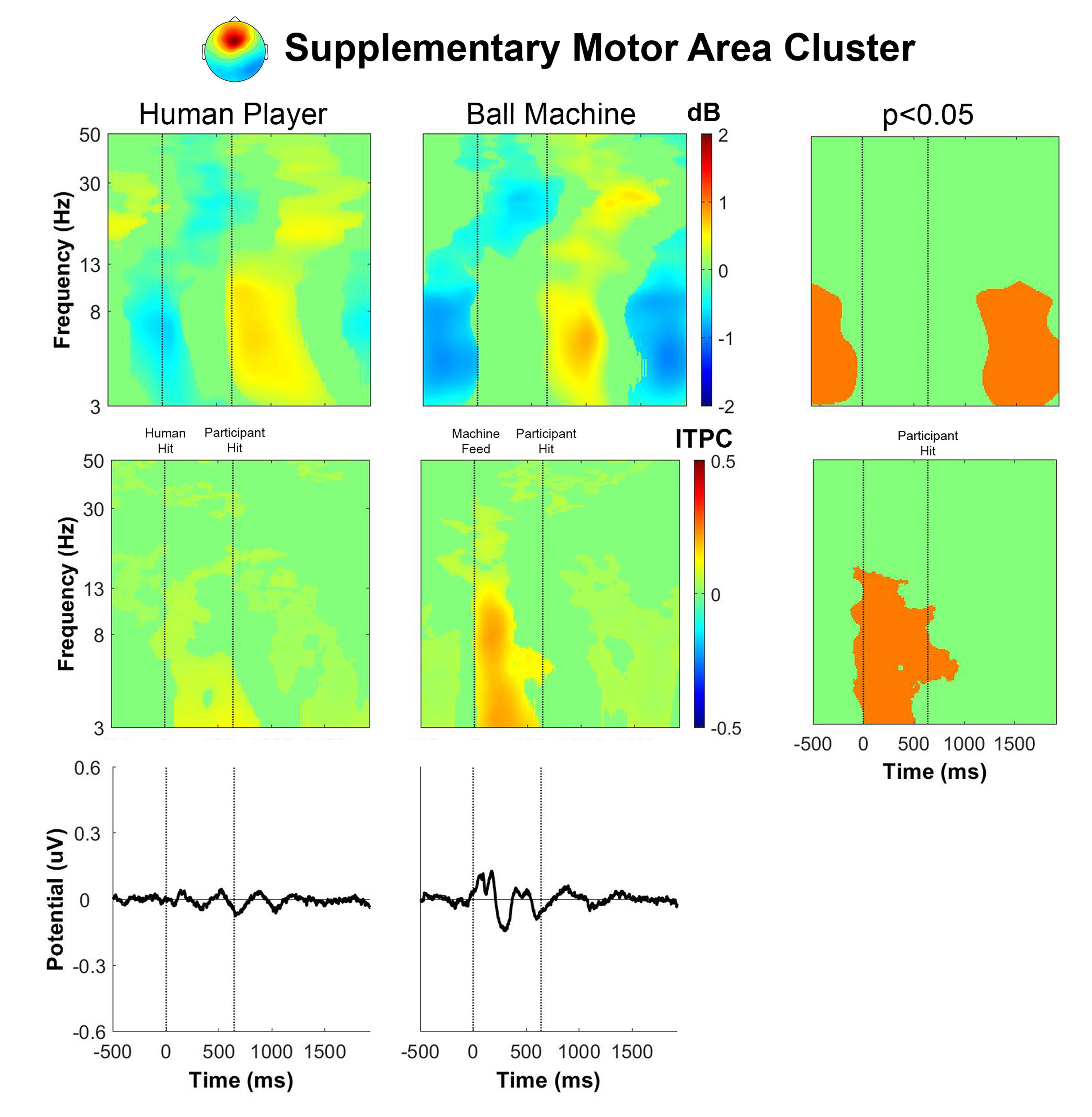

Supplement: Extended Data Figure 9-3 — Supplementary motor area cluster group average (n = 15) results. Download Figure 9-3, TIF file. [file enu-eN-NWR-0463-22-s08.tif]

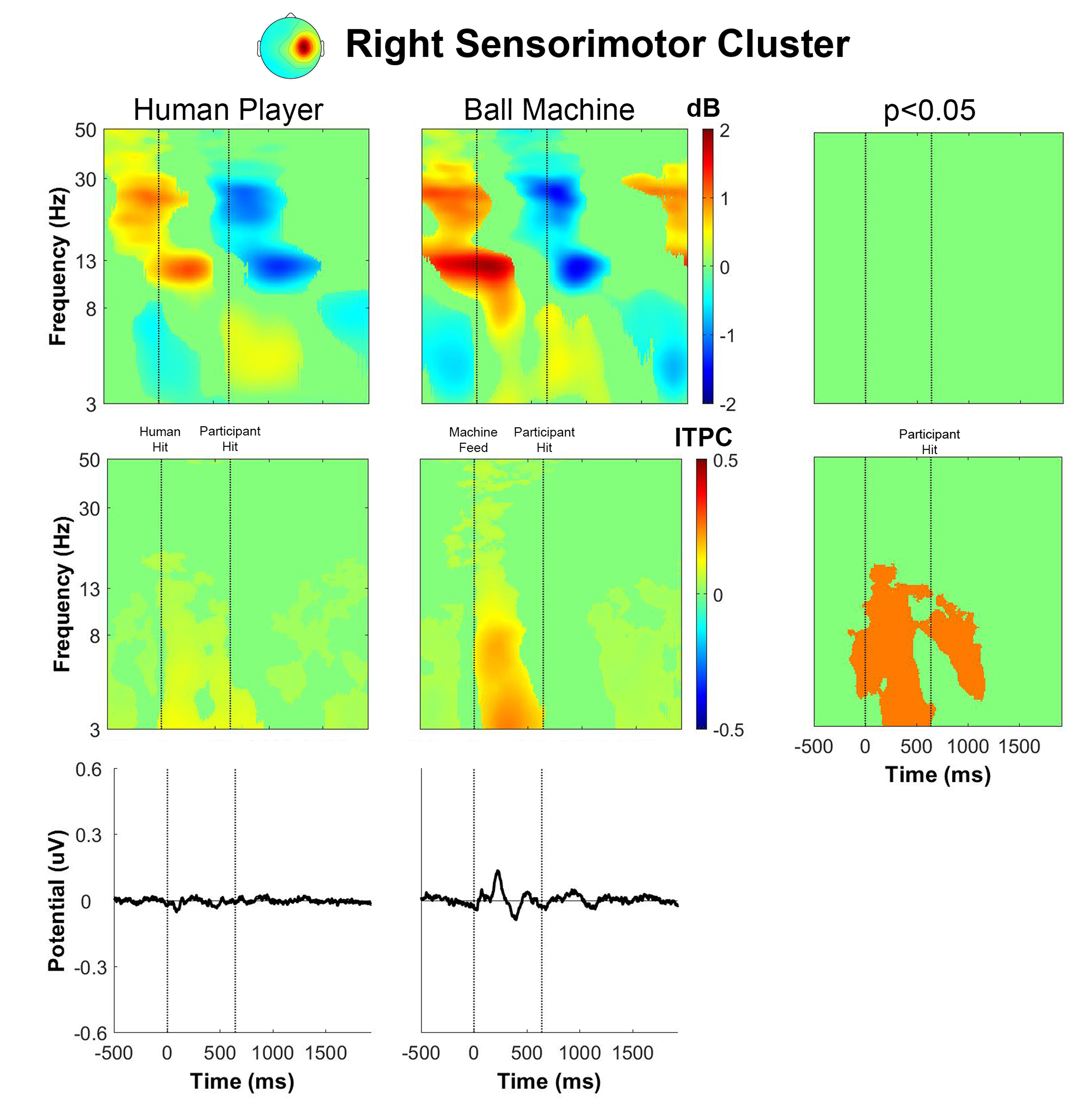

Supplement: Extended Data Figure 9-4 — Right sensorimotor cluster group average (n = 13) results. Download Figure 9-4, TIF file. [file enu-eN-NWR-0463-22-s09.tif]

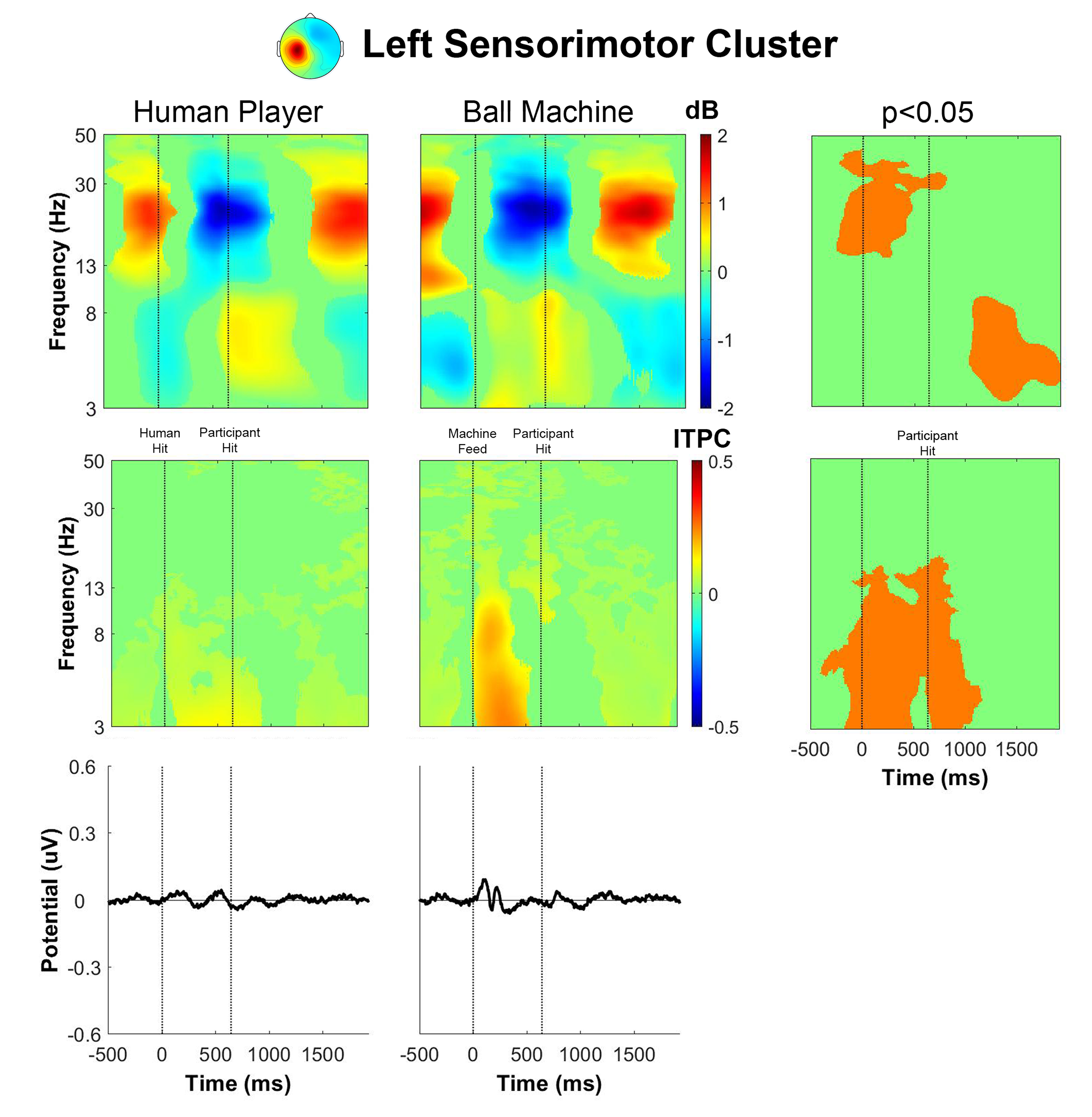

Supplement: Extended Data Figure 9-5 — Left sensorimotor cluster group average (n = 17) results. Download Figure 9-5, TIF file. [file enu-eN-NWR-0463-22-s10.tif]
